# The role of autophagy in spinal cord injury: Mechanisms, crosstalk, and therapeutic strategies

**DOI:** 10.4103/NRR.NRR-D-24-01467

**Published:** 2025-05-06

**Authors:** Rui Wang, Zhen Niu, Runze Tian, Aini Chen, Huangmei Liao, Rui Kuang, Ying Feng, Guangyu Chin, Jiesheng Xie, Ping Zhu, Chi Teng Vong, Ge Li

**Affiliations:** 1 Guangdong Provincial Key Laboratory of Pathogenesis, Targeted Prevention and Treatment of Heart Disease, Medical Research Institute, Guangdong Provincial People’s Hospital (Guangdong Academy of Medical Sciences), Southern Medical University, Guangzhou, Guangdong Province, China; 2 School of Basic Medical Sciences, Southern Medical University, Guangzhou, Guangdong Province, China; 3 School of Medicine, South China University of Technology, Guangzhou, Guangdong Province, China; 4 Sub-specialty Diagnosis Research Center of Neurological Disorder, Guangzhou Huayin Medical Laboratory Center, Guangzhou, Guangdong Province, China; 5 Guangzhou Key Laboratory of Cardiac Pathogenesis and Prevention, Guangdong Provincial People’s Hospital (Guangdong Academy of Medical Sciences), Southern Medical University, Guangzhou, Guangdong Province, China; 6 State Key Laboratory of Quality Research in Chinese Medicine, Institute of Chinese Medical Sciences, University of Macau, Macao Special Administrative Region, China; 7 Macau Center for Research and Development in Chinese Medicine, University of Macau, Macao Special Administrative Region, China

**Keywords:** apoptosis, autophagy, chaperone-mediated autophagy, ferroptosis, macroautophagy, microautophagy, neuronal protection, parthanatos, pyroptosis, spinal cord injury

## Abstract

Spinal cord injury is a neurological disorder resulting from trauma, typically affecting sensory and motor function at the injury site, even leading to paralysis and internal dysfunction. The treatment of spinal cord injury mainly relies on pharmacological and surgical interventions; however, significant challenges remain in the protection and repair of neural tissues. Autophagy, an intracellular process responsible for the degradation and recycling of macromolecular components, plays a vital role in spinal cord injury, alleviating the severity of injury by inhibiting cell apoptosis and inflammatory responses. In this review, we provide an overview of the physiological mechanisms underlying autophagy and spinal cord injury and detail the crosstalk between autophagy and other modes of cell death in spinal cord injury. In addition, we discuss the potential of targeting autophagy as a therapeutic strategy for spinal cord injury through approaches that focus on promoting or inhibiting this process, targeting specific autophagic substrates or pathways, and combining autophagy modulation with other neuroprotective or restorative interventions. In summary, this review proposes that strict regulation of autophagy may represent a viable strategy for the treatment of spinal cord injury.

## Introduction

Spinal cord injury (SCI) is a damaging neurological and pathological condition resulting from either traumatic or non-traumatic causes (Anjum et al., 2020). Traumatic SCI primarily includes traffic accidents, falls, sports injuries, and industrial accidents. Non-traumatic SCI, meanwhile, encompasses spinal cord tumors, spinal cord infections, ischemic injuries, degenerative diseases of the spinal cord, degenerative changes in the vertebrae, autoimmune diseases, drugs or toxins, and spinal cord hypoxia. The consequences of SCI are often severe, frequently leading to the loss of movement and sensation at the site of injury and potentially also paralysis and impaired internal organ function. Due to age-related bone changes, the prognosis of SCI is substantially worse for older patients than younger ones (Wang et al., 2025a). However, the prognosis of SCI also depends on other factors, such as the location and severity of the lesion. Injuries to the cervical spine can result in quadriplegia in mild cases and death following severe damage. In contrast, injuries at the thoracic and lumbar spine often lead to paraplegia or the loss of function of the corresponding organ or limb (Kwakkel and Dobkin, 2021; Vasankari et al., 2022). Over recent years, advancements in medical research have significantly improved the survival rate of patients with traumatic SCI (Gadot et al., 2022). Current treatment options for SCI mainly include drug therapy and surgery. Surgical treatment aims to relieve acute spinal cord compression and the inflammatory environment at the site of injury, while pharmacological intervention mainly focuses on relieving pain and managing clinical symptoms (Fehlings et al., 2022). However, repairing neural tissue and restoring neuronal function following SCI remain considerable challenges in the treatment of this condition.

Autophagy, also known as type II programmed cell death, refers to the process by which damaged, aged, or excess biomolecules and organelles are selectively degraded in lysosomes, releasing small molecules for cellular recycling (Cao et al., 2021; Trelford and Di Guglielmo, 2021). The maintenance of proteostasis and organelle integrity through autophagy is essential for cellular homeostasis and cell viability (Debnath et al., 2023). Research on autophagy has advanced significantly since its initial observation in the 1950s (Appelmans et al., 1955; Rahman and Wright, 1975; de Duve, 2005). A study has also indicated that autophagy can serve as a key therapeutic target for several diseases (Mizushima and Levine, 2020). Both the induction and over-activation of autophagy are frequently observed in SCI, suggesting that targeting this process may serve as a strategy for neuroprotection and repair in the injured spinal cord. Here, we comprehensively review the physiological processes of autophagy, traumatic SCI, and the regulation of autophagy after SCI. We also discuss the potential of targeting autophagy as a therapeutic intervention for SCI, aiming to provide a novel strategy for the treatment of this condition.

## Search Strategy

In this narrative review, we discussed the role of autophagy in treating SCI. More than 95% of the cited literatures were written in English, and more than 60% of the cited literatures were published in the last 3 years. Literatures were collected from July to October 2024, and the search engines used include PubMed, China National Knowledge Infrastructure (CNKI), Wanfang, and Web of Science. The following keywords were used to search the relevant literature: autophagy, physiological process of autophagy, type of autophagy, key molecules of autophagy, spinal cord injury, physiological process of spinal cord injury, treatment of spinal cord injury, autophagy and spinal cord injury.

## Autophagy

Autophagy is a biological mechanism that plays an important role in the processes of cellular metabolism and death. It can be defined as the process of degrading substances within lysosomes/vesicles in cells, and the resulting metabolic macromolecular components are recycled in the tissues (Trelford and Di Guglielmo, 2021; Li et al., 2024a).

### Evolutionary history of autophagy

**Figure 1:**
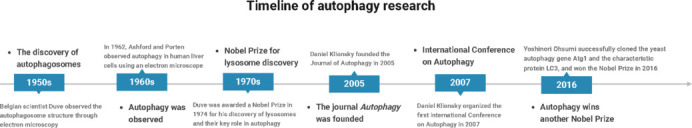
Theoretical development process and milestone events of autophagy. In the 1950s, the Belgian scientist Christian de Duff observed the structure of autophagy through an electron microscope and first proposed the concept of “autophagy.” In 1974, he received the Nobel Prize in Physiology or Medicine for his discovery of lysosomes. In 1997, Otsu Yoshimori cloned the first yeast autophagy genes, Atg1 and LC3, which promoted the study of autophagy in yeast models. In 2001, Nobutomo Mizushima, a Professor of Biochemistry and Molecular Biology at the University of Tokyo, reported the function of Atg5, which was considered the first step in the study of molecular mechanisms in mammals. In 2003, a uniform naming convention was established on the basis of autophagy-related genes in yeast, in which the letters “ATG” and “autophagy” were used to distinguish different genes. In 2005, the biochemist Daniel Krionsky founded the first journal on autophagy. In 2016, a research team from the University of Tokyo reported that Atg13 plays an important regulatory role in the autophagy initiation complex. Created with BioRender.com. ATG: Autophagy-related gene; LC3: light chain 3.

The study of autophagy has won two Nobel Prizes in Physiology or Medicine. The first awarded to the Belgian scientist Christian de Duve was the Nobel Prize in 1974 for the discovery of lysosomes, which play important roles in the mechanism of cellular autophagy (Appelmans et al., 1955). Second, the Japanese team led by Yoshinori Osumi was awarded the Nobel Prize in 2016 for its exploration of the initiation mechanism of cellular autophagy. The exploration of autophagy can be divided into the following stages (**Figure 1**).

#### Early insights and discoveries (1950s–1960s)

In 1955, Christian de Duve, a Belgian biochemist, discovered lysosomes, which are cellular organelles involved in the breakdown of macromolecules. While studying the digestive activities of cells, de Duve proposed that the degradation of cellular components might be mediated by these lysosomes (Appelmans et al., 1955). In 1963, Christian de Duve coined the term “autophagy” (from the Greek “auto” = self, and “phagy” = eating) to describe the process by which cells degrade and recycle their components, particularly in response to stress or nutrient deprivation (de Duve, 2005).

#### Electron microscopy and early observations (1970s–1980s)

In 1974, researchers used an electron microscope to look at autophagic vacuoles in cells. These vacuoles were identified as double-membrane structures involved in the degradation of cytoplasmic components. In 1975, studies on liver cells revealed the first evidence of autophagosomes, which are double-membrane vesicles that fuse with lysosomes to degrade their contents. During the 1970s and 1980s, the idea emerged that autophagy might serve as a way for cells to survive under conditions of nutrient deprivation. Researchers have also hypothesized that autophagy could be involved in cellular homeostasis, maintaining the balance of organelles and proteins in cells (Rahman and Wright, 1975).

#### Molecular understanding and gene discovery (1990s)

In the early 1990s, in 1993, yeast (Saccharomyces cerevisiae) became the model organism for studying autophagy. The autophagy-related genes (Atg)1, Atg5, and Atg7 were identified as essential genes in the autophagic process. These genes were discovered through genetic screens in yeast, marking the beginning of understanding the molecular machinery behind autophagy (Takeshige et al., 1992). In 1997, the discovery of Atg8, a protein that binds to autophagic vesicles (autophagosomes), helped researchers understand how these vesicles are formed and how they are targeted to lysosomes for degradation. This was crucial for the stepwise model of autophagosome formation (Ichimura et al., 2000). In 1999, the discovery of light chain 3 (LC3), a mammalian homolog of yeast Atg8, provided a key marker for monitoring autophagic flux. LC3 is involved in the extension of the autophagosome membrane and is widely used as a diagnostic tool in an autophagy study (Kabeya et al., 2000).

#### Autophagy and disease mechanisms (2000s)

From 2000–2005, research focused on the role of autophagy in various diseases. In neurodegenerative diseases, studies have revealed that autophagy is critical for clearing damaged proteins and organelles, and defects in autophagy pathways are implicated in diseases such as Parkinson’s disease (PD), Alzheimer’s disease (AD), and Huntington’s disease. Mutations in Atg genes or defects in autophagic processes lead to the accumulation of toxic aggregates in neurons (Ravikumar et al., 2004). In cancer, autophagy has a dual role. On one hand, autophagy can prevent cancer by removing damaged cells; on the other hand, it can promote the survival of cancer cells by helping them adapt to nutrient-starved environments, which is often the case in solid tumors (Edinger and Thompson, 2003). In 2003, the target of rapamycin (TOR) pathway was identified as a major negative regulator of autophagy. Mammalian target of rapamycin complex 1 (mTORC1) inhibits autophagy in response to nutrients, growth factors, and energy levels. In contrast, when nutrients are scarce, autophagy is activated to provide the cell with essential building blocks (Scott et al., 2004). In 2005, Daniel Klionsky founded the first journal on autophagy, *Autophagy*, and published a series of research papers on the mechanisms of autophagy. Beclin-1, a key autophagy regulator, has been identified in humans. It interacts with phosphoinositide-3-kinase (PI3K) class III complexes to promote autophagy. The role of Beclin-1 in both tumor suppression and autophagic regulation has opened new therapeutic avenues for cancer (Yue et al., 2003). In 2007, Daniel Klionsky organized the first International Conference on Autophagy, and his research advanced the field of autophagy.

#### Nobel Prize and major advances (2010s)

In 2010, rapamycin, an inhibitor of mammalian target of rapamycin (mTOR), was discovered to induce autophagy. This discovery led to studies exploring its potential as a therapeutic agent for neurodegenerative diseases (such as AD) and cancer. The ability of rapamycin to stimulate autophagy has significant implications for aging and longevity (Stephan et al., 2010). In 2016, Yoshinori Ohsumi was awarded the Nobel Prize in Physiology or Medicine for his pioneering work on the molecular mechanisms of autophagy. Ohsumi identified key genes involved in autophagy in yeast and established the basic machinery for this process. His discoveries, including the identification of Atg genes, were groundbreaking and provided insight into the regulation of autophagy in higher organisms, including humans.

#### Autophagy and therapeutic strategies (2020s)

In the last few years of the 2020s, the therapeutic potential of autophagy has been increasingly studied in various fields:

Aging and longevity: Autophagy has been implicated in aging, with increasing evidence that enhancing autophagy could extend lifespan by reducing the accumulation of damaged proteins and organelles. Studies have focused on autophagy-modulating drugs, such as rapamycin and “spermidine,” and their potential to slow aging and prevent age-related diseases (Hofer et al., 2024). For cancer therapy, researchers are exploring the possibility of targeting autophagy, either by inhibiting autophagy to prevent cancer cells from using it for survival or by enhancing it to help remove damaged or mutated cancer cells (Debnath et al., 2023). Increasing evidence suggests that autophagy plays a protective role in neurodegenerative diseases. For example, enhancing autophagy may help clear protein aggregates that characterize diseases such as AD and PD (Li et al., 2023a). In cardiovascular disease, autophagy also plays a critical role in heart health, particularly in response to stress. Researchers are studying ways to activate autophagy in cardiac cells to improve heart function, particularly after heart attack (Tian et al., 2025). After 2023, the ongoing development of autophagy enhancers (e.g., Beclin-1 activators, torin-2, hydroxychloroquine, metformin) was part of a broader effort to modulate this process for therapeutic purposes. Researchers are also exploring autophagy-related biomarkers for diagnosing and monitoring diseases and therapeutic responses (Jain et al., 2023).

The in-depth research and development of autophagy is not limited to the normal regulation of cellular autophagy in the body. An increasing number of autophagy pathways in disease modes have been discovered, and their key molecular targets have gradually been identified, laying the foundation for targeted autophagy in disease treatment.

### Classification of autophagy

**Figure 2:**
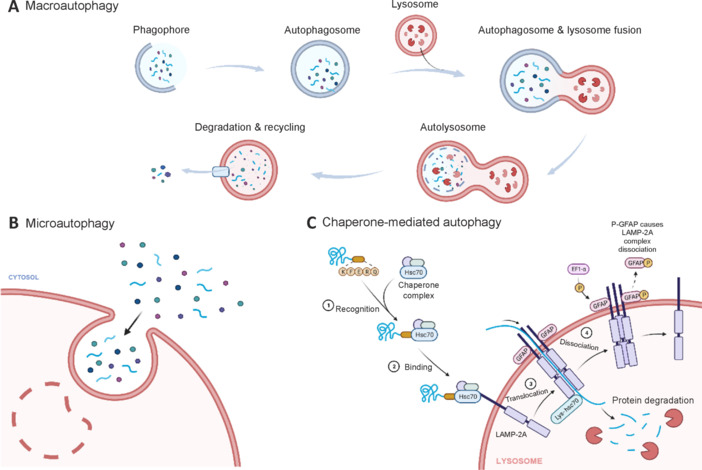
Three types of autophagy. (A) Macroautophagy: Autophagosomes form and fuse with lysosomes to degrade substrates. Macroautophagy is a highly conserved lysosomal degradation pathway that sequesters substrates into double-membrane vesicles called autagosomes and delivers them to lysosomes or vacuoles for degradation and recycling. (B) Microautophagy: Direct wrapping of free substrates by lysosomes. Vesicles containing organelles or inclusions interact directly with and fuse with lysosomes, and microautophagy is more selective than macroautophagy and can be triggered by signaling molecules on the surface of damaged organelles. (C) Chaperone-mediated autophagy: Vesicles containing organelles or inclusions interact directly with and fuse with lysosomes, and microautophagy is more selective than macroautophagy and can be triggered by signaling molecules on the surface of damaged organelles. A channel is formed on the surface of the lysosome. Substrate proteins with specific pentapeptide sequences bound to Hsc70 are shuttled through the channel to enter the lysosome and are subsequently degraded by lysosomal enzymes. Created with BioRender.com. EF1-α: Elongation factor one alpha; GFAP: glial fibrillary acidic protein; Hsc70: heat shock cognate 71 kDa protein; LAMP-2A: lysosomal-associated membrane protein 2A.

On the basis of the different ways in which substrates are transported from the cytoplasm to the lysosomal lumen, autophagy can be divided into three forms: macroautophagy, microautophagy and chaperone-mediated autophagy (CMA). Macroautophagy is a standard process of autophagy, and the degraded substrate is encased in a double-membrane vesicle called the autophagosome, which fuses with the lysosome to degrade the substrate. Unlike macroautophagy, microautophagy involves direct phagocytosis of cytoplasmic components (proteins and organelles) by lysosomes or late endosomes without the involvement of autophagosomes. CMA is also a lysosomal degradation pathway, but its primary degradation is based on enveloping proteins that contain specific pentapeptide motifs. The primary process of CMA involves the recognition, binding, unfolding and translocation of proteins assisted by molecular chaperones from the cytoplasm to the lysosome (**Figure 2**; Tedesco et al., 2023).

#### Macroautophagy

Macroautophagy is a highly conserved lysosomal degradation pathway (Antonioli et al., 2017) that sequesters substrates into double-membrane vesicles called autagosomes and delivers them to lysosomes or vacuoles for degradation and recycling (Huang and Guo, 2024). Macroautophagy occurs at a basal level in virtually all eukaryotic cells and is commonly upregulated in response to cellular stress caused by diverse physiological stimuli, including nutrient deprivation, withdrawal of growth factors, and oxidative and mechanical stress, as pathological conditions, including drug and radiation therapy (Filali-Mouncef et al., 2022).

Upon receiving the induction signal for macroautophagy, cells form a small, vesicle-like membranous structure at a certain location in the cytoplasm, which then continuously expands, similar to a bowl made of two layers of lipid bilayers, engulfing damaged, aged, or excess biomacrom and organelles. This bowl-like structure can be observed under an electron microscope and is called the phagophore (Kraft and Reggiori, 2024), which is one of the hallmarks of autophagy. The phagophore extends continuously, engulfing any components in the cytoplasm, including organelles, and then becomes the autophosome (Polyansky et al., 2022; Tsong et al., 2023; Zhen and Stenmark, 2023). The observation of autophagosomes under an electron microscope is the second piece of irrefutable evidence for the occurrence of autophagy. After the formation of autophagosomes, they fuse with the cell’s endocytosed phagosomes, pinosomes, and terminate. The autophagosome fuses with the lysosome to form the autolysosome (Tian et al., 2021), during which the inner membrane of the autophagosome is degraded by lysosomal enzymes, the contents of the two are combined, and the “cargo” in the autophagosome is also degraded. The products (such as amino acids and fatty acids) are transported to the cytoplasm for reuse by the cell, while the residue is either expelled from the cell or remains in the cytoplasm (Shen et al., 2021).

**Figure 3:**
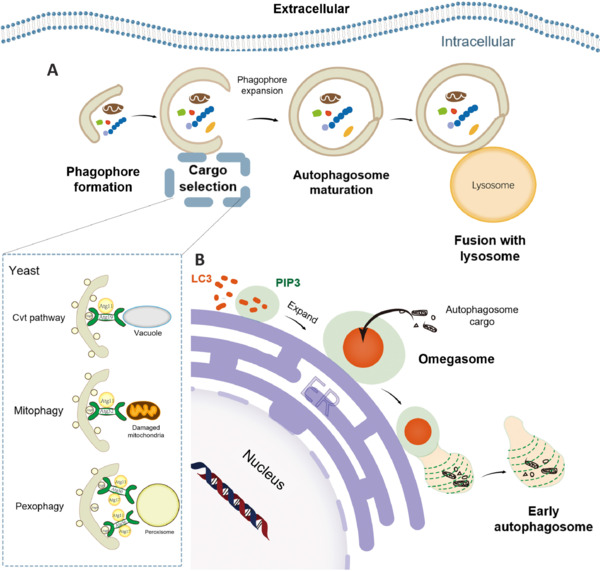
Two mechanisms of autophagosome formation in macroautophagy and three mechanisms of selective autophagy in macroautophagy. (A) Formation of autophagosomes through the transport of membranes from different types of organelles. (B) During amino acid starvation, vesicles interact with the ER, forming PtdIns3P (PI3P) on the membrane that binds to the ER. This membrane domain binds with autophagosome proteins (red), thus creating a hybrid membrane structure (omegasome). The autophagosome membrane buds inward when it expands to the maximum size, and early double-membrane autophagosomes are formed. Cvt pathway: Cytoplasm to vacuole-targeting pathway; ER: endoplasmic reticulum; PIP3: phosphatidylinositol-3,4,5-trisphosphate.

Phagosome formation is the first step in the initiation of macroautophagy. There are at least two mechanisms by which autophagosomes can be formed. One mechanism is similar to that of yeast, where autophagic vesicles are formed by transporting membranes of different organelles (**Figure 3A**). The omegasome is another mechanism that uses an omega-shaped membrane structure or cradle stem from the phosphatidylinositol 3-phosphate (PtdIns3P) of the endoplasmic reticulum (ER). The omegasome is an organelle consisting of a PtdIns3P lipid membrane bilayer associated with the autophagic process. It is a subfield of ER and has a form similar to the Greek capital letter omega (Ω). Omegasomes can act as precursors for autophagosome formation by recruiting proteins that generate ring extensions on the ER membrane (**Figure 3B**; Molinari, 2021; Wenzel et al., 2022; Li et al., 2023b). In addition, phagosome membrane formation in yeast occurs in the presence of an Atg protein locus. At the phagophore assembly site, phagosomes can be formed and mediated by Atg proteins assembled at predetermined locations (Yin et al., 2020; Gurunathan et al., 2021). However, Atg proteins have multiple localization sites in mammals, and no yeast phagophore assembly sites have been identified in mammalian cells (Hollenstein and Kraft, 2020). Research on Atg genes is the predominant method for studying the formation of phagophores and autophagosomes. In addition, macroautophagy can be classified into nonselective and selective autophagy (Li et al., 2021b; Rubio-Tomás et al., 2023). Selective autophagy involves different mechanisms for different cargoes, and three main mechanisms have been well studied, including the cytoplasm-to-vacuole targeting pathway for resident hydrolases, mitophagy for damaged mitochondria, and pexophagy for unwanted peroxidases. In the process of the cytoplasm-to-vacuole targeting pathway, Atg19 and Atg34 are receptors, and Atg11 is the scaffold. Atg32 is the receptor during mitophagy, and Atg11 is the scaffold (Gubas and Dikic, 2022; Riaz et al., 2024). In addition, Atg30 and Atg36 are receptors, and Atg11 and Atg17 are scaffolds in the pexophagy process (**Figure 3**).

At the beginning of autophagy, adenosine 5′-monophosphate (AMP)-activated protein kinase (AMPK) promotes autophagosome production by inhibiting the formation of the mTORC1 complex, weakening the inhibitory effect of mTORC1 on the formation of the Unc51-like autophagy activating kinase 1 (ULK1) complex (Zhu et al., 2022). Moreover, the activated kinase c-Jun N-terminal kinase (JNK) disrupts Beclin-1/BCL-2 and Beclin-1/Bcl-2-interacting mediator (BIM) complexes by phosphorylating BCL2 and BIM, releasing free Beclin-1 (Nahata et al., 2021). The released Beclin-1 activates vascular protein sorting 34 (VPS34) and combines with it to form a complex, and the PtdIns3P produced promotes the extension of the autophagosome (Ohashi, 2021). ATG5, ATG12, and autophagy-related 16-like 1 (ATG16L) undergo a series of reactions to form the AT5-ATG12-ATG16L polymeric complex, which then fuses with the autophagosome. The cysteine protease ATG4 cleaves LC3 into LC3-I, which is subsequently processed by ATG3, AT7, and phosphatidylethanolamine to form LC3-II. LC3-II is then inserted into the autophagosome (Li et al., 2020a). Syntaxin 17 (STX17) combines with soluble N-ethylmaleimide-sensitive factor attachment protein receptor 29 kDa (SNAP29) and vesicle-associated membrane protein 8 (VAMP8) to form a soluble NSF attachment protein receptor (SNARE) complex, which is then transferred to the autophagosome membrane, where it allows lysosomes to fuse with autophagosomes and form autolysosomes (Tian et al., 2021). Eventually, the cellular materials are degraded into small molecules by lysosomes and recycled.

#### Microautophagy

Vesicles containing organelles or inclusions interact directly with and fuse with lysosomes, and microautophagy is more selective than macroautophagy and can be triggered by signaling molecules on the surface of damaged organelles (Yamamoto and Matsui, 2024). Like macroautophagy, in mammals, microautophagy can also be divided into two types, selective and nonselective microautophagy, depending on the way in which the cargo is engulfed. Nonselective autophagy is initiated under starvation and stress conditions, whereas selective autophagy is initiated during the clearance of damaged organelles. In addition, microautophagy can also be divided into lytic and fusion autophagy, depending on how the cargo that is engulfed into the interior of the lysosome is processed. Cleaved microautophagy refers to the membrane cleavage of lysosomes after engulfing cargo, which wraps the cargo into the internal vesicles and is degraded internally by hydrolases. Fused microautophagy refers to the membrane extension and expansion of lysosomes to wrap cargo, followed by the formation of internal vesicles, which are degraded by hydrolytic enzymes (**Figure 2B**; Wang et al., 2023b).

In yeast, three main types of selective microautophagy are involved: micromitophagy, microreticulophagy and micronucleophagy. All three types of microautophagy are fusion types of microautophagy, and their molecular mechanisms are activated by the recognition of specific receptors. The specific receptors for micromitophagy include Bcell lymphoma 2 (Bcl-2), Bcl-2-interacting protein 3 (BNIP3), and BNIP3-like (BNIP3L), also known as Nip3-like protein X (NIX) and FUN14 domain-containing protein 1 (FUNDC1) (Onishi et al., 2021). The initiation of micromitophagy plays an important role in regulating cellular metabolism, monitoring mitochondrial mass and exerting antioxidant activities (Choubey et al., 2021; Tong et al., 2022). Moreover, specific receptors for phagocytosis include peroxisome autophagy-specific protein 2 (Paz2, a Pichia pastoris homolog of the wine yeast Aut7/Apg8) and Atg9. Microcytosis can regulate lipid metabolism and maintain and detect peroxidase levels (Phyo et al., 2022). The specific receptors for micronucleophagy are cyclic guanosine 5′-monophosphate-AMP synthase (cGAS) and lysosomal-associated membrane protein 2C (LAMP2C). They are activated in response to DNA damage and regulate the expression of many genes (Zhao et al., 2021a; Wang et al., 2023b). In addition, microautophagy, which is a pathway of selective wrapping of mitochondria and nuclear fragments or aggregation by multivesicular bodies and transporting them into lysosomes for degradation, is also mediated by multiple vesicular bodies. The formation of multivesicular bodies requires the involvement of the endosomal sorting complex required for transport (eSCRT) complex and VPS4ATPase (**Additional Table 1**; Huotari and Helenius, 2011; Vietri et al., 2020; Gurunathan et al., 2021).

**Additional Table 1:** Different types of microautophagy

Type	Sign	Cargo	Function
Non-selective	-	Random cytoplasm, vacuole membrane	Emergency turnover of cellular materials
Selective	BINP3, NIX, FUNDC1	Mitochondria	Monitoring mitochondrial mass; regulation of cellular metabolism; resistance to oxidative stress
	Paz2, Atg9	Peroxisomes	Regulation of lipid metabolism; detection of peroxidase
	cGAS, LAMP2C	Nucleus fragments	In response to DNA damage; regulation of gene expressions
	SQSTM1/p62, NBR1, OPTN	Aggregated ubiquitinated proteins	Monitor the removal of abnormal proteins
	Mutivesicular bodies	Mitochondria, nuclear fragments or aggregates	Exosome production

BINP3: Brain injury-derived neurotrophic peptide 3; cGAS: cyclic guanosine 5'-monophosphate-AMP synthase; FUNDC1: FUN14 domain-containing protein 1; LAMP2C: lysosomal-associated membrane protein 2C; NBR1: neighbor of BRCA1 gene 1; NIX: Nip3-like protein X; OPTN: optineurin; Paz2: peroxisome autophagy specific protein 2; SQSTM1: sequestosome 1, p62.

#### Chaperone-mediated autophagy

Cytoplasmic proteins bind to molecular chaperones and are transported into the lysosomal lumen, where they are digested by lysosomal enzymes. Unlike the first two types of autophagy, CMA does not use vesicles, is highly selective (Liu et al., 2023), and often involves the chaperone protein heat shock cognate 71 kDa protein, which recognizes target proteins with a unique five-residue motif (KFERQ-like) for lysosomal degradation. The protein LAMP2A on the lysosomal membrane recognizes the KFERQ motif exposed by the binding protein, “guiding” the target protein into the lysosomes for degradation (Liao et al., 2021b; Yang et al., 2023; Yao and Shen, 2023).

The processes of CMA differ from those of microphages and macrophages. CMA can be considered a selective autophagy pathway, and its primary substrate is proteins (Wang et al., 2020a; Choubey et al., 2021). The phagocytosis of macrophages requires the formation of autophagosomes, and the phagocytosis of macrophages is related to the membrane morphology of lysosomes. The degradation process of proteins by CMA can be divided into four steps: (a) recognizing and binding to the substrate proteins; (b) positioning and transporting of the substrate proteins; (c) passing through the lysosomal membrane; and (d) lysosomal degradation (Cuervo and Wong, 2014; Muthukottiappan and Winter, 2021). Studies have shown that the substrate protein has a membrane-penetrating effect and directly enters lysosomes through a “channel” formed on the lysosome membrane (**Figure 2C**; Salvador et al., 2000; Ji et al., 2022).

The death of neurons is related primarily to the degradation of proteins, so the mishandling of abnormal proteins often leads to the deposition of proteins (Fricker et al., 2018). Therefore, CMA plays both positive and negative roles in neurodegeneration. For example, in AD, the wild-type tau protein binds to LAMP2A as a substrate protein of CMA, but LAMP2A cannot completely transport the tau protein into lysosomes, which results in the deposition of the tau protein on lysosomal membranes and thereby leads to lysosome breakage and leakage (Wang et al., 2009; Kanno et al., 2022).

#### Others

On the basis of whether the degraded substrate is specific, autophagy can be divided into selective microautophagy and nonselective microautophagy. In selective microautophagy, autophagosomes selectively degrade specific cellular components through autophagy (Zellner et al., 2021). On the basis of the selectivity of autophagy for the degradation of different substrates, autophagy can be divided into mitophagy, reticulophagy, ribophagy, pexophagy, etc. This form of autophagy is particularly important when cells respond to specific stresses or injuries (Liu et al., 2024). Nonselective microautophagy refers to the process in which there is no obvious selectivity in the degradation of substrates, potentially degrading various cells simultaneously. This type of autophagy is random in the degradation of substrates; for example, during nutrient deprivation, cells randomly take up cytoplasmic material for degradation (He, 2022).

### Markers of autophagy

Autophagy participates in various hepatic metabolic processes, providing amino acids, free fatty acids, and glucose for energy production and the biosynthesis of new molecules in starved cells, while autophagy also controls the quality and quantity of cellular organelles such as mitochondria (Li et al., 2023d). Moreover, autophagy can identify and remove damaged organelles such as mitochondria, as well as misfolded or aggregated organelles, preventing their accumulation within the cell and thus avoiding toxic effects on the cell, thus playing an important role in the development and differentiation of cells (Sun et al., 2023). Autophagy participates in innate and adaptive immune responses, protecting the body from infection and maintaining environmental stability and immune homeostasis by clearing pathogens and cells (Zheng et al., 2023). Autophagy disorders are associated with the occurrence and development of neurodegenerative diseases such as PD and AD (Jiao et al., 2025; Song et al., 2025). Enhancing autophagy might slow the progression of these diseases (Li et al., 2024b). There are three markers of autophagy: (a) LC3 on the autophagosome membrane, which is a characteristic marker of autophagy. In the absence of autophagy, the LC3 synthesized in the cell is processed and converted into LC3-I, which is soluble in the cytoplasm and is expressed constitutively. During autophagy, LC3-I is converted to LC-II, which is associated with the autophagosome membrane; the conversion of LC3-I to LC3-II is essential for the formation of autophagosomes, and an increase in the ratio of LC3-II or LC3-II/LC3-I indicates the formation of activated autophagosomes after SCI (Shi et al., 2018). (b) The Beclin-1 protein, another autophagy-related protein, plays an important role in the formation of autophagosome precursors (Fujita et al., 2015). An increase in the expression levels of the *Beclin-1* gene and protein indicates an increase in autophagy after SCI. (c) The p62 protein, a receptor for autophagic degradation substrates, includes ubiquitinated protein aggregates targeted for elimination. The p62 protein can form targets for autophagosomes through ubiquitination. During autophagy, the p62 protein is degraded along with its substrates; therefore, the p62 protein can represent autophagic flux, with reduced P62 protein expression indicating increased autophagy levels (Tanabe et al., 2011).

### Mechanisms of autophagy

#### Activation of autophagy

Under non-pathological conditions, autophagy is present in most cell types (Wang et al., 2025b; Zheng et al., 2025). Autophagy is typically triggered by stress-related signals, both inter- and extracellular, such as starvation, growth factor deprivation, hypoxia, energy expenditure, endoplasmic reticulum (ER) stress, immune signaling, infection, cell surface receptor signaling, and bacterial toxins (Cao et al., 2021). Two main regulatory pathways are known to be induced by these stimuli, namely, mTOR and PI3K (Xu et al., 2020; Cao et al., 2021).

mTOR is a serine/threonine kinase mainly involved in nutrient state perception and autophagy during cell growth (Glick et al., 2010; Seibert et al., 2021). mTOR forms two multiprotein complexes, mTORC1 and mTORC2. The former plays a role in promoting cell growth and metabolism and can directly activate protein kinase B (AKT1), while the latter indirectly promotes mTORC1 signaling and functions as a regulator of the cytoskeleton (Szwed et al., 2021). mTOR is regulated by insulin signaling, growth factor receptor signaling, adenosine triphosphate (ATP) levels, and hypoxia (Wu and Storey, 2021).

PI3K is a phosphoinositide 3-kinase and a key signaling molecule that primarily participates in the regulation of cell growth, metabolism, and autophagy. It activates its downstream AKT signaling pathway by catalyzing the formation of phosphatidylinositol-3,4,5-trisphosphate (PIP3). The regulation of autophagy by the PI3K pathway reflects the dynamic balance between anabolic and catabolic metabolism within cells. Under nutrient-rich conditions, the PI3K pathway preferentially inhibits autophagy, thereby promoting cell growth and proliferation. In contrast, under conditions of energy deficiency or stress, the PI3K pathway is inhibited, leading to the activation of autophagy, which allows cells to degrade endogenous components in response to environmental changes (Soto-Avellaneda and Morrison, 2020; Xu et al., 2020).

#### Formation of autophagosomes

Once autophagy has been triggered, autophagosomes gradually form and expand through the coordinated activity of a series of protein complexes. The inhibition of mTORC1 leads to the activation of the ULK1 complex, which is responsible for the initiation of autophagosome formation. The components of the ULK1 complex include ATG13, FIP200, and ATG101, key regulatory factors in the initiation of autophagy (Nazio et al., 2013; Xiang et al., 2020). Additionally, the PI3K complex, comprising VPS34, VPS15, beclin-1, and ATG14, can generate phosphatidylinositol-3-phosphate (PI3P), which aids in the recruitment of other autophagy-related proteins to the autophagosome membrane (DebBurman et al., 1995; Soto-Avellaneda and Morrison, 2020).

### Extension and expansion of autophagosomes

#### The ATG12-ATG5-ATG16L complex

This complex plays an important role in membrane extension, assisting LC3 in binding to phosphatidylethanolamine, yielding LC3-II. This process is crucial for the extension of the autophagic membrane.

#### LC3

LC3 aids in the bending and stabilization of membranes, promoting the amplification of autophagosomes through its association with autophagic membranes (Li et al., 2020a).

#### Identification and packaging of autophagic cargo

During autophagosome expansion, specific substances in the cytoplasm are selectively enveloped, including damaged organelles, misfolded proteins, and intracellular pathogens. A key factor involved in this selection process is p62 (SQSTM1), an autophagy receptor that recognizes and binds to ubiquitinated targets and interacts with LC3-II to facilitate the entry of these targets into the autophagosome (Tanabe et al., 2011). Other selective autophagy receptors, such as NBR1 (Gomez-Sanchez et al., 2015) and optineurin, also participate in specific autophagic processes, aiding in the recognition of particular cargo.

#### Fusion of autophagosomes and lysosomes

The autophagosome ultimately fuses with the lysosome, forming an autolysosome. Subsequently, hydrolases within the lysosome degrade the contents of the autophagosome. Some of the factors known to be involved in autophagosome/lysosome fusion include (1) Rab7, is a small GTPase that participates in guiding the movement of autophagosomes towards lysosomes (Bai et al., 2019; Lin et al., 2021); (2) SNARE proteins, which mediate membrane fusion between autophagosomes and lysosomes (Tian et al., 2021); and (3) V-ATPases, which regulate the acidic environment of the autophagosome by pumping protons into it, thus creating conditions for subsequent membrane fusion (Sun et al., 2023).

#### Regulation and feedback mechanisms of autophagy

Autophagy is a highly regulated process with multi-layered feedback mechanisms. mTOR kinases inhibit autophagy via two mechanisms. One involves the control of ULK1 translation and transcription through the regulation of downstream signaling factors. In the other, mTOR mediates the formation of autophagosomes by directly regulating ATG proteins (Kim et al., 2011; Xiang et al., 2020). The main mechanism underlying the cascade of mTOR-controlled downstream signaling factors is the crosstalk between mTORC1, ULK1, and AMPK (Dunlop and Tee, 2014; Shi et al., 2021a). mTORC1 inhibits ULK1 both by directly phosphorylating it and via the phosphorylation of activating molecule in BECN1-regulated autophagy protein 1 (AMBRA1). In contrast to mTORC1, AMPK activates ULK1. The antagonism between AMPK and mTORC1 plays a vital homeostatic role in the regulation of autophagy. The PI3K pathway also negatively regulates autophagy. This inhibitory mechanism is triggered by ULK1-dependent phosphorylation of AMBRA1, which results in the release of the AMBRA1/ATG14/beclin-1/VPS34 complex from the dynein motor complex and its translocation to the ER (Nazio et al., 2013; Kaur and Changotra, 2020). PI3K-I activation allows VPS34 to catalyze the conversion of phosphatidylinositol to PI3P, forming a signaling platform for autophagosome initiation. PI3Ps bind to AKT and phosphoinositide-dependent kinase, leading to AKT activation and the inhibition of autophagy (DebBurman et al., 1995; Soto-Avellaneda and Morrison, 2020).

## Spinal Cord Injury

**Figure 4:**
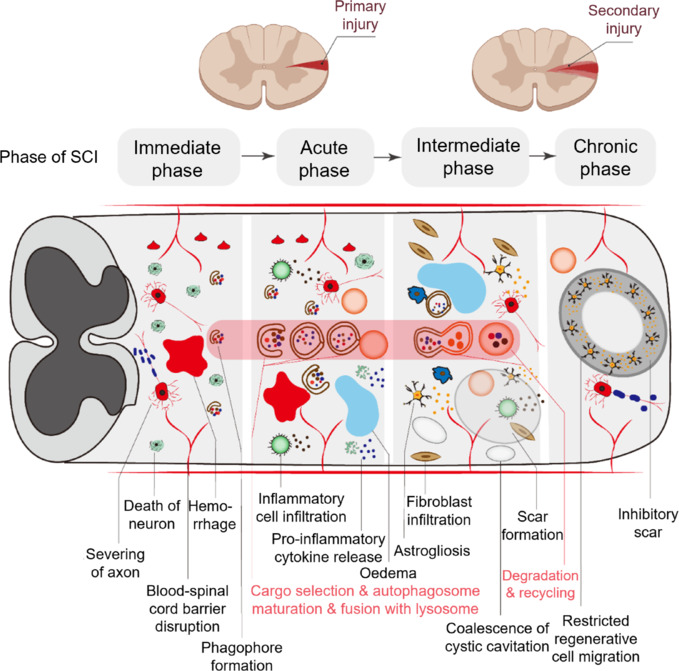
Pathophysiological processes in SCI. SCI can be divided into the immediate, acute, intermediate and chronic phases. The acute phase is often accompanied by an inflammatory response, disturbed ion homeostasis and excitotoxicity. Apoptosis, oxidative stress and demyelination reactions occur in the intermediate phase. In the chronic phase, myelin regeneration and scarring occur. In the immediate phase, the initial mechanical trauma to the spinal cord initiates disruption of the BSCB, severing of axons, hemorrhage, and death of neurons and glia when phagophores begin to form. The acute phase initiates a secondary injury cascade, which is characterized by inflammatory cell infiltration, edema, and proinflammatory cytokine release, when autophagosomes mature and fuse with lysosomes. In the subacute and intermediate phases, persistent ischemia and inflammatory cell infiltration can further lead to the formation of cystic microcavities and cell death, which is accompanied by the simultaneous proliferation of astrocytes and fibroblasts. In the chronic phase, astroglial scars mature and enlarge, thus restricting cell migration. SCI: Spinal cord injury.

Spinal cord fracture is a severe form of SCI, resulting in the displacement of the pyramidal nerve or the protrusion of bone fragments into the spinal canal, thereby damaging the spinal cord or cauda equina nerve to varying degrees. SCI can be classified based on injury severity (incomplete SCI, complete SCI, and spinal cord concussion) or by the stage of injury (primary and secondary) (Zha, 2025). The direct destruction of spinal cord structure by broken bones or other invasive objects is categorized as primary SCI, which is generally accompanied by the destruction of spinal cord parenchyma, the axonal network, and glial membranes, in addition to bleeding. The degree of primary injury can directly affect the occurrence and development of secondary injury. Further SCI progression results from an inflammatory reaction, edema, glial scar formation secondary to SCI, and apoptosis. The continuous accumulation of intracellular calcium (Ca^2+^) ions and increases in glutamic acid and reactive oxygen species (ROS) concentrations can lead to neuronal excitotoxicity and the consequent destruction of nucleic acids, proteins, and phospholipids, ultimately resulting in neurological dysfunction (**Figure 4**; Anjum et al., 2020). Multiple mechanisms are involved in the development of secondary SCI.

### Vascular changes

These involve a reduction in the spinal microvascular, vasospasm, blood-brain barrier disruption, and bleeding, which result in edema and increased intramedullary pressure (Kaptanoglu et al., 2003; Cao et al., 2015; Zhang et al., 2019).

### Free radical formation

Between 60 to 150 minutes after spinal cord ischemia, the levels of arachidonic acid oxidation, catalyzed by free radicals and cyclooxygenase, are significantly increased (Basu et al., 2001; Freyermuth-Trujillo et al., 2022; Hao et al., 2024), accompanied by the generation of monoamine oxidase-B byproducts, the auto-oxidation of biogenic amine neurotransmitters, the activation of xanthine oxidase, and hemoglobin oxidation. Meanwhile, SCI also leads to microglia, macrophage, and neutrophil activation, resulting in the continuous production of oxygen free radicals (O_2_^–^; Hall, 2011). Besides, given that the central nervous system is rich in iron, iron-dependent hydroxyl radicals can quickly form during secondary injury (David et al., 2022). Lipid peroxidation is often accompanied by the ferrous or ferric ion-catalyzed formation of iron and lipid hydroperoxides (Kontogianni and Gerothanassis, 2022). Lipid peroxidation can also destroy cell and organelle membranes (Hall, 2011).

### Disruption of ion balance

In secondary SCI, the balance of sodium (Na^+^), potassium (K^+^), and Ca^2+^ ions can be disrupted. The distribution of K^+^ channels is also changed, which hinders axon conduction. The upregulation of Na^+^ channel expression can increase intracellular Ca^2+^ concentrations, while the opening of Ca^2+^ channels causes neurotoxicity and white matter damage.

### Apoptosis

SCI is accompanied by the apoptosis of neurons, microglia, oligodendrocytes, and astrocytes. The programmed death of neurons usually occurs rapidly, followed by the apoptosis of oligodendrocytes. The apoptosis of microglia peaks on day 8 after injury. Apoptosis can be detected in active tissues adjacent to the injury center, potentially explaining why the damaged area continues to gradually expand (Shi et al., 2021b).

### Inflammatory reaction

SCI activates an inflammatory cascade, in which cells such as leukocytes, macrophages, astrocytes, and microglia are activated in the nerve tissues. M1-type macrophages produce ROS and interleukin (IL)-2 to promote inflammatory responses and remove damaged tissues, while M2-type macrophages downregulate ROS production, thereby antagonizing the inflammatory reaction as well as promoting cell proliferation and the elimination of apoptotic cells. M2 macrophages can also promote remyelination and reduce axon withering (Gensel and Zhang, 2015). Furthermore, astrocytes produce chemokines and recruit inflammatory cells after injury, and can also promote the regeneration of M1 macrophages. Astrocytes participate in inflammatory reactions through the signal transducer and activator of transcription 3 (STAT3) pathway. The phosphorylation of STAT3 can promote scar formation and thus limit the expansion of inflammation (Constantinescu et al., 2005; Cekanaviciute and Buckwalter, 2016; Fu et al., 2022; Zhang et al., 2023b). Following SCI, microglia are also recruited, and they accumulate adjacent to the injured area rather than in the injury center. They secrete neurotrophin-3, nerve growth factor, and thrombochondroitin, which promotes the repair of injury (Chamak et al., 1994).

### Excitotoxicity

After SCI, ischemia, inflammation, free radicals, and other factors cause neurons and glial cells to release a large amount of the neurotransmitter glutamate, which can activate α-amino-3-hydroxy-5-methyl-4-isoxazolepropionic acid glutamate receptors in white matter. This leads to Ca^2+^ influx and intracellular Ca^2+^ overload, resulting in the activation of a range of signaling pathways, such as protein kinase, nuclear factor-kappa B (NF-κB), and mitochondrial pathways, eventually leading to oligodendrocyte apoptosis and axonal degeneration. The apoptosis of oligodendrocytes can further aggravate demyelination and induce axonal rupture, resulting in the loss of white matter function (Park et al., 2004; Fu et al., 2022).

## Autophagy and Spinal Cord Injury

Autophagy in the spinal cord plays a crucial role in maintaining cellular homeostasis and contributing to repair processes. Under physiological conditions, autophagic activity in neurons is relatively low (Lipinski et al., 2015; Nikoletopoulou et al., 2015). Nonetheless, autophagy in these cells serves as a key pathway for the metabolic degradation of neuronal components, both under physiological conditions and in response to neural tissue injury. Similarly, basal autophagic activity in astrocytes is generally minimal under normal conditions. Other cell types in the spinal cord, such as microglia and oligodendrocytes, also engage in autophagy as part of their physiological functions. However, the mechanisms and implications of autophagy in these cell types are poorly understood, highlighting the need for additional studies.

Autophagy contributes to the pathogeneses of many neurodegenerative conditions, such as Alzheimer’s disease (AD), Parkinson’s disease (PD), and Huntington’s disease, and is also involved in traumatic SCI. The continuous accumulation of intracellular Ca^2+^ ions along with increases in glutamic acid levels and ROS concentrations can lead to neuronal excitotoxicity, which destroys nucleic acids, proteins, and phospholipids, ultimately leading to neurological dysfunction (Anjum et al., 2020). The damaged cellular components resulting from trauma may be necessary for the induction of autophagy. Many studies have demonstrated that autophagic proteins such as beclin-1 and LC3-II are highly expressed at and around the injury area after SCI. Autophagy plays an equally important role in both stages (primary and secondary) of SCI (Guha et al., 2023). However, whether the induction of autophagy is inhibited after SCI may depend on the type of traumatic injury. Astrocytes are more sensitive to ischemic and hypoxic injury than neurons. Ischemia and hypoxia stimulate a conversion from basal to elevated levels of autophagy in astrocytes, which can further damage these cells (Xu and Zhang, 2011). Autophagy is inhibited in minor contusions and severe compression injuries (**Figure 4**; Whittemore et al., 2022).

### Autophagy in neurons, astrocytes, oligodendrocytes, microglia, and Schwann cells

Neurons are typically composed of cell bodies, dendritic branches, and axons. According to reports, an increase in autophagy in neurons usually occurs in the early stage of SCI, and neurons are the first cells in which LC3 upregulation is detected after SCI. In normal conditions, the level of autophagy in neurons is relatively low (Lipinski et al., 2015; Nikoletopoulou et al., 2015). Changes in neuronal autophagy may be SCI model- and neuronal subtype-dependent. For instance, it was reported that spinal cord neurons are more susceptible to the inhibition of autophagy flux than brain neurons, while motor neurons exhibit greater susceptibility to autophagy flux disruption than sensory neurons (Wu and Lipinski, 2019). Additionally, distinct forms of autophagy have been identified in neurons—non-selective autophagy in the distal axon, aggrephagy in the cell body, ERphagy, and mitophagy. Autophagosome formation has been detected in the distal axon after SCI, as evidenced by the observed retrograde transport of acidified vesicles from the axon to the soma (Hollenbeck, 1993; Stavoe and Holzbaur, 2019).

Muñoz-Galdeano et al. (2018) reported that astrocytes in all regions of the undamaged spinal cord lack detectable LC3 staining, except for those at the interface with the pia mater. This suggests that autophagic flux in spinal cord astrocytes is very low or absent under physiological conditions. However, 7 days post-SCI, astrocytes near the site of injury accumulate autophagic structures, and exhibit strong staining of beclin-1, suggesting that autophagy is overactive in these glial cells. Their location at the edge of the injury and their strong glial fibrillary acidic protein staining suggest that autophagic is particularly highly activated in reactive astrocytes. Similar activation has previously been observed in several central nervous system injury models, such as spinal cord hemisection (Kanno et al., 2011), nerve lumbar axotomy (Zhang et al., 2013a), and traumatic brain injury. The activation of autophagy may serve as a protective mechanism against the harmful conditions faced by astrocytes in the penumbra during exposure to nutrient deprivation and inflammation (Korenić et al., 2015). Ischemia and hypoxia induce an increase in the levels of autophagy in astrocytes, which can further damage these cells. In a model of permanent cerebral venous occlusion, the protein and mRNA levels of LC3-II and cathepsin B were reported to be increased, and autophagosome and autophagolysosome formation was enhanced, which exacerbated astrocyte injury (Whittemore et al., 2022). However, it has also been demonstrated that autophagy activation under ischemic-hypoxic conditions can protect astrocytes by delaying apoptosis and necrosis (Kasprowska et al., 2017; Zhou et al., 2020).

Mature oligodendrocytes express the autophagy marker LC3-II at high levels. Autophagy involving ATG5 is required for the formation of myelin sheaths. When nerve cells and myelin sheaths are damaged, oligodendrocytes initiate autophagy to promote their own survival as well as for myelin repair (Bankston et al., 2019). In a rat SCI model, it has been demonstrated that the intravesical injection of neurotrophin-3 inhibited the expression of beclin-1 and other autophagy proteins in oligodendrocytes and promoted their proliferation (Cong et al., 2020).

Following acute injury to the spinal cord, microglia are rapidly activated as part of the inflammatory response. This activation plays a vital role in the repair of the injury and the reconstruction of the injury microenvironment. The initiation of autophagy in microglia balances their activation levels and controls their activation state (Su et al., 2016; Ray, 2020). Studies have shown that autophagy in microglia at the injured area becomes dysregulated by day 3 after spinal cord damage, leading to the production of pro-inflammatory cytokines by these cells (Fan et al., 2016; Li et al., 2022). Increasing the level of microglial autophagy can reduce inflammation and contribute to the repair of damaged tissue (Li et al., 2022).

After SCI, Schwann cells migrate from the peripheral nervous system to the lesion site in the spinal cord. SCI may promote Schwann cell autophagy. Demyelination stimulates the activation of the ULK complex, members of the ATG9 recycling system, and the autophagy-related ATG7 gene. Meanwhile, LC3-II levels peak 5 days after injury and the autophagic substrate neighbor of BRCA1 gene 1 (NBR1) is rapidly catabolized. Autophagosomal structures surrounding myelin fragments can also be observed by electron microscopy (Gomez-Sanchez et al., 2015). Exosomes secreted by Schwann cells can help protect axons after SCI by increasing autophagy levels and reducing apoptosis through the epidermal growth factor receptor/AKT/mTOR signaling pathway (Pan et al., 2022).

### Alterations in autophagy levels after spinal cord injury

The initiation of autophagy is one of the causes of neuronal death following SCI. Relatively severe primary damage may lead to autophagic flux hyperactivation, resulting in the death of autophagic neurons during traumatic secondary SCI (Lipinski et al., 2015; Liao et al., 2021a). In a rat model of secondary SCI, rats treated with valproate displayed improved Basso-Beattie-Bresnahan scale scores, an increase in the number of motor neurons in the abdominal horn, and reduced myelin injury at 42 days post-injury compared with vehicle-treated animals. This demonstrated that valproate enhanced motor function (Hao et al., 2013) by reducing autophagy. Excessive autophagy enhances caspase-1 activation through a non-classical ATG5-dependent pathway, thus promoting inflammasome activation and increasing IL-1β and IL-18 synthesis and secretion. Moreover, excessive autophagy can promote the secretion of these proinflammatory factors into the cytoplasm, aggravate the inflammatory damage in tissues, and cause autophagic cell death (Li et al., 2022). Excessive autophagy promotes beclin-1/Bcl-2 complex dissociation and excessive production of ROS in p53 signaling, which leads to the production of free Bcl-2-associated X protein (BAX), thus affecting axon regeneration and the restoration of body nerve function (Chen et al., 2012). In a recent study, the initiation of autophagy was often observed in secondary SCI. Autophagy can be assessed in different cells of the spinal cord through the detection of specific biomarkers or by direct observation of autophagosomal structures (Luo and Tao, 2020). These biomarkers mainly include beclin-1, LC3-II, and p62. In a mouse SCI model, the beclin-1 protein level was significantly increased at the injury site. This increase began 4 hours after injury, peaked on day 3, and continued until day 21. Moreover, high beclin-1 expression was detected not only in neurons but also in astrocytes and oligodendrocytes (Sung and Jimenez-Sanchez, 2020; Wang et al., 2023a). Similarly, in mice, LC3-II was found to be highly expressed in the SCI region, paralleling beclin-1 expression. Moreover, LC3-II expression was detected in the cell nucleus, suggestive of the presence of autophagic cells, consistent with electron microscopic observations. These findings indicated that autophagy is activated in injured neural tissues after SCI (Kanno et al., 2011; Liao et al., 2021a). LC3-II upregulation commenced 4 hours after SCI in neurons, but only after 3 days in astrocytes (Hou et al., 2014; Wang et al., 2021b). Employing a rat model of SCI (spinal cord impact contusion), Chen et al. (2012) confirmed the presence of autophagy and the progression of autophagic cell death after SCI by analyzing LC3 expression at 2 and 4 hours, and 1, 3, and 7 days after SCI, as well as assessing changes in cellular localization and ultrastructure (Chen et al., 2012). Moreover, the p62 protein is involved in the degradation of autophagic substrates and is also a substrate for autophagy. After SCI, the level of p62 gradually increases, indicating that autophagy is impaired, and that impaired degradation in cells can lead to the accumulation of autophagic structures (Liu et al., 2015a; Muñoz-Galdeano et al., 2018; Zhou et al., 2020). In a model of chronic spinal cord compression, p62 expression was noted to be negatively correlated with the number of neurons. In white matter, p62 moved caudally to the site of compression as demyelination and axon degeneration progressed (Forston et al., 2023). Autophagy can be triggered early in SCI, and the level of autophagy changes over time. This indicates that autophagy is a significant factor influencing the progression and development of secondary SCI. The specific manifestations are as follows: (1) Scar formation. The formation of glial scars, primarily composed of microglia, macrophages, extracellular matrix, and astrocytes, is an important mechanism underlying SCI pathology. SCI is followed by macrophage infiltration into the site of injury and microglial activation, which leads to the local activation of astrocytes and, consequently, the formation of glial scars, which affects synaptic plasticity. It has been demonstrated that rapamycin enhances the inhibition of the mTOR signaling pathway and activates autophagy, thereby blocking the release of presynaptic membrane neurotransmitters. This implies that autophagy is a homeostatic mechanism related to the synaptic microenvironment (Todorova and Blokland, 2017). (2) Demyelination and remyelination. An imbalance in the local microenvironment in the spinal cord after SCI and the death of oligodendrocytes may be the main causes of demyelination. (Blight, 1985) showed that the rate of oligodendrocyte apoptosis at the center of the lesion peaked within 1 week of SCI, leading to local demyelination; however, the myelin sheath of uninjured axons surrounding the lesion remained almost intact. Three months after SCI, the damaged axons appeared to remyelinate (Blight, 1985). An imbalance between demyelination and remyelination leads to mechanical injury and local ischemia, which, in turn, promotes the release of inflammatory cytokines, oxidative stress, excitotoxicity mediated by glutamate and ATP, and autophagy. A decline in the expression of beclin-1, LC3, and glutathione peroxidase leads to a decrease in mitochondrial autophagy, the disruption of cellular homeostasis, growth cone collapse, axonal retraction, and an increase in the risk of apoptosis (Alizadeh et al., 2015). (3) Inflammatory response. Inflammation plays a significant role in secondary injury following SCI. Research has shown that NF-κB upregulates the expression of several genes, such as tumor necrosis factor-alpha (TNF-α) and IL-1β, which exacerbate secondary injury after SCI. Autophagy plays a similarly important regulatory role in inflammatory signaling, primarily via the modulation of inflammatory transcriptional responses. In autophagy-deficient cells after SCI, the level of the adaptor protein p62 is upregulated, and the consequent reduction of autophagy promotes the activation of kinases, thereby increasing the NF-κB-mediated production of TNF-α and IL-1β (Sebastian-Valverde and Pasinetti, 2020). In addition, the relationship between autophagy and the heterogeneity of neural tissue cells also merits some consideration. The levels of autophagy vary among astrocytes, neurons, microglia, oligodendrocytes, and Schwann cells owing to their diverse functions.

Moreover, the level of autophagy, that is, whether it is enhanced or weakened, is currently determined by the detection of autophagy flux. The term “autophagic flux” describes the dynamic nature of autophagy, encompassing the entire process. This includes the formation of autophagosomes; their subsequent maturation, fusion with lysosomes, and degradation; and the redistribution of toxic proteins and damaged organelles back into the cytosol (Zhang et al., 2013a). Autophagic flux is important for intracellular “refresh,” that is, the maintenance of homeostasis, which is particularly important for the health of terminally differentiated cells, such as neurons and oligodendrocytes (Mizushima, 2025). A growing body of literature supports that lysosomal defects or the failure of fusion between autophagosomes and lysosomes leads to the blockade of autophagic flux in neurons that underlies the development of several central nervous system disorders (Nikoletopoulou et al., 2015). Three classical approaches—dynamic LC3 turnover, p62 degradation, and the degradation of long-lived proteins—are used to assess autophagic flux by directly measuring the degradation of autophagic substrates in lysosomes. The detection of p62 degradation by western blotting or immunofluorescence is relatively convenient and is the most widely used of the three methods for tracking autophagic flux in a range of diseases, including SCI (Liu et al., 2015a). Autophagic flux blockade is associated with increased p62 levels, while enhanced autophagic flux results in the opposite effect (Mizushima, 2025).

#### Autophagy and traumatic spinal cord injury

Using the NYU-Impactor is a well-established method for inducing contusive SCI. The apparatus consists of a drop rod that weighs 10 g and has an impact surface of 2 mm. When the rod is released from a height of 12.5 mm, it induces moderate contusive injury in rats. After contusive SCI, infiltrating macrophages over-engulf myelin sheaths and cellular debris, and are transformed into lipid-rich, foamy macrophages. Ryan et al. (2024a) noted that the macrophage-specific inhibition of PI3K using liposomes significantly reduced foamy macrophage numbers at the site of injury after mid-thoracic contusive SCI in mice. RNA sequencing and *in vitro* analysis of foamy macrophages further showed that autophagy was increased while phagocytosis was decreased after PI3K inhibition. These observations suggest that the formation of pro-inflammatory foamy macrophages after SCI is due to the activation of PI3K signaling, which promotes phagocytosis and inhibits autophagy. Chen et al. (2024) showed that [D-Ala2, D-Leu5]-enkephalin (DADLE), a selective agonist of the delta opioid receptor, significantly increased autophagic flux and inhibited necroptosis in a mouse spinal cord contusion model. Concurrently, DADLE restored autophagic flux by decreasing lysosomal membrane permeability. Further analysis showed that DADLE partially reversed the inhibition of lysosomal membrane permeability and necroptosis by reducing cytosolic phospholipase A2 phosphorylation and promoting autophagy, preventing lysosomal enzymes and other hydrolytic enzymes from leaking from the lysosomal cavity into the cytoplasm.

Using a hemisection model of SCI, Kanno et al. (2011) studied autophagic activity in damaged nerve tissue after SCI from the perspective of cytochemistry and anatomy. Hemisection was performed at T10 in adult female rats. The authors found that the levels of LC3 and beclin-1 at the injury site were significantly increased 4 hours after hemisection injury, peaking on day 3, and persisting at the site of injury for 21 days. LC3 and beclin-1 positivity was observed in neurons, astrocytes, and oligodendrocytes. Electron microscopic observation showed that the formation of autophagic vacuoles was increased in damaged cells. The nuclei of LC3-expressing, terminal deoxyribonucleotide transferase-mediated nick-end labeled cells were round and consistent with autophagic cell death, without shrinking and fragmentation as observed in apoptotic nuclei, suggesting that autophagy is significantly activated and autophagic cell death is induced in damaged neural tissue after SCI.

Wang et al. (2015b) assessed autophagic activity in a rat model of oppressive SCI and showed that the LC3-II/LC3-I ratio and beclin-1 levels were significantly increased after SCI, peaking on day 3, and decreasing slightly on day 7. Xu et al. (2023) demonstrated that edaravone reduced blood-spinal cord barrier disruption and promoted functional recovery after SCI. This effect may be attributed to its role in preserving angiogenic activity, enhancing autophagy, and promoting the phosphorylation and mutual negative interactions among receptor-interacting kinase 1/receptor-interacting kinase 3/mixed lineage kinase (RIP1/RIP3/MLKL).

Fang et al. (2016) investigated autophagy in rats with ischemia-reperfusion SCI (IRSCI), which was induced via the clamping of the thoracic aortic arch for 14 minutes. The results showed that both the LC3-II/LC3-I ratio and the expression of beclin-1 were increased after IRSCI, and displayed two peaks, one at 8 hours and the other at 72 hours post-injury, and then slowly decreased to baseline. In a separate study, Wei et al. (2016) induced IRSCI by compressing the thoracic aortic arch by balloon inflation for 10 minutes. They reported that the expression of LC3-II and beclin-1 began to increase 3 hours after IRSCI, peaked at 24 hours, and was still high at the 48-hour sampling time point. Tam et al. (2024) investigated the role of autophagy in the maintenance of cell homeostasis using ATG7 knockout mice subjected to ischemia-reperfusion injury complemented by ATG7 knockout in a H9c2 cardiomyoblast cellular model exposed to hypoxia-reoxygenation. The results confirmed that ATG7 knockout led to autophagy (including mitophagy) deficiency. Additionally, ATG7 knockout H9c2 cells subjected to hypoxia and reoxygenation exhibited increased cell death compared with wild-type cells. Notably, the authors found that autophagy deficiency increased stress-induced mitochondrial fission, the release of mitochondrial DNA, and sterile inflammation, namely, the activation of the stimulator of interferon genes/interferon regulatory factor 3 (STING/IRF3) axis, leading to elevated interferon-α expression. Muñoz-Galdeano et al. (2018) summarized the autophagic response after SCI in mice, focusing on cell-specific changes in autophagy following spinal cord contusion, as detailed in **Additional Table 2**.

**Additional Table 2:** Overview of the autophagic response after SCI

Animal	Injury	Analyses	Result	Reference
Wistar rats	Hemisection at T8	IF of Beclin-1, Atg 5, Atg7 and ULK1 at 30 mpi, 6 hpi, 1, 14, and 42 dpi	IF: increase of initiation markers in damaged axons at 1 dpi; increase of LC3 puncta at 14 dpi	Ribas et al., 2015
	Weight-drop contusion 10 g-25 mm at T10	qPCR of Beclin-1 and LC3 at 1, 2, 6, 24, 48 and 72 hpi; IB and IF of Beclin-1 and LC3 at 2 hpi	qPCR: increase of LC3 and Beclin-1 transcription at 2 hpi; IB and IF: increase of protein levels of LC3 and Beclin-1 at 2 hpi	Wang et al., 2015b
SD rats	Weight-drop contusion 10 g-25 mm at T9	IB of LC3 at 2, 4 hpi, 1, 3, and 7 dpi; IF of LC3 and TEM at 2 hpi	IB: increase of LC3-II at 2 hpi-1 dpi; IF/TEM 2 hpi: LC3 and autophagosomes in neurons; absent in astrocytes	Chen et al., 2012
	15 g vascular clip compression at T9	IB of LC3 and p62 at 7 dpi; IF of LC3 at 7 dpi	IB: increase of LC3-II plus decrease of p62 at 7 dpi; IF: increase of LC3 at 7 dpi, no cell type data	Zhang et al., 2013b
	Weight-drop contusion 10 g-25 mm at T8	IB and IF of LC3, Beclin-1, and p62 at 1, 7, 14, and 35 dpi	IB: increase of LC3-II and p62 at 1 dpi; no changes in Beclin-1; Blockage of autophagy confirmed by markers of lysosomes IF: blockage in neurons at 1 dpi; in microglia and oligodendrocytes at 1/7 dpi. No changes in astrocytes	Liu et al., 2015a
	Weight-drop contusion 10 g-25 mm at T10	IB and qPCR of LC3 and Beclin-at 1, 2, 6, 24, 48, and 72 hpi; IF of LC3 at 2 hpi	qPCR and IB: increase of protein and mRNA expression of LC3-II & Beclin-1 at 2 hpi; IF: increase of LC3 signal in neurons at 2 hpi	Hao et al., 2013
	Weight-drop contusion 10 g-25 mm at T9	IB of LC3 and Beclin-1 at 12 hpi, 1, 3, 7, 14 and 21 dpi	IB: increase of LC3-II at 3-7 dpi; increase of Beclin-1 at 12-24 hpi	Zhang et al., 2014
	30 g force clip compression in T7-T10	IB of Beclin-1, LC3 and p62 at 3, 7, and 14 dpi; IF of LC3 + NeuN at 7 dpi	IB: increase of LC3-II/LC3-I at 7 dpi, increase of Beclin-1 at 14 dpi; Decrease of p62 at 3, 7 dpi; IF: LC3 staining in motoneurons at 7 dpi	Wang et al., 2017
	Ligation of L5 spinal nerve	IF of LC3 and Beclin-1 at 14 dpi	IF: increase of LC3 and Beclin-1 in neurons at 14 dpi; Slight increase in astrocytes, no changes in microglia and oligodendrocytes	Zhang et al., 2013a
	Hemisection between T9/T10	IF of LC3 and Beclin-1 at 4, 8 hpi, 1, 3, 7 and 21 dpi; IB of LC3, Beclin-1 and p62 at the same times; RT-PCR of Beclin-1 at the same times	IB: increase of LC3 and Beclin-1 peaking at 3 dpi; gradual decrease of p62 RT-PCR: Beclin-1 expression increases from 4-8 dpi and remains overexpressed; IF: neurons follow the IB pattern; astrocytes undergo a strong increase at 3 dpi. No data on oligodendrocytes or microglia	Hou et al., 2014
	Weight-drop contusion of 10 g-30 mm at T9-T10	IB of Beclin-1, LC3 and p62 at 7 dpi	IB: increase of LC3-II, decrease of p62, and slight increase of Beclin-1 at 7 dpi	Zhao et al., 2017
C57BL/6J mice	Hemisociion at T10	IF of LC3 at 4 hpi, 1, 3, 7 and 21 dpi; IB of LC3 and TEM at 3 dpi	IF: increase of LC3 staining at 3 dpi among neurons, astrocytes and oligodendrocytes Confirmed by IB and TEM	Kanno et al., 2011
	Hemisection at T10	IF of Beclin-1 at 4 hpi, 1, 3, 7 and 21 dpi; IB of Beclin 1 and TEM at 3 dpi	IF: increase of Beclin-1 staining at 3 dpi among neurons, astrocytes and oligodendrocytes Confirmed by IB & TEM	Kanno et al., 2009
	Weight-drop contusion 10 g-3 mm at T10	IB of Beclin-1 and LC3 at 1 and 3 dpi; IF of Beclin-1 and LC3 at 3 dpi	IB: increase of LC3-II & Beclin-1 at 1 or 3 dpi IF: increase in the number of LC3 and Beclin-1 positive cells at 3 dpi	Sekiguchi et al., 2012
	Hemisoction at T12	IB of Becin-1 and p62 at 4 dpi	IB: increase of LC3-II at 4 dpi; no changes of Beclin-1 expression	Goldshmit et al., 2015
White rabbits	Ischemia (15 min in right femoral artery)	IB and IHC of LC3 at 8 hpi, 1 and 2 dpi	IB: peak of LC3-II at 8 hpi; IF: LC3 staining in motorneurons at 8 hpi	Fujita et al., 2015

dpi: Day(s) post injury; hpi: hour(s) post injury; IB: immunoblot; IF: immunoluorescene; mpi: minute(s) post injury; qPCR: quantitative real-time polymerase chain reaction; RT-PCR: reverse transcription-polymerase chain reaction; TEM: trarsmission electron microscopy; ULK1: Unc51-like autophagy activating kinase 1.

#### Autophagy and degenerative spinal cord injury

Cervical myelopathy (DCM), amyotrophic lateral sclerosis (ALS), and multiple sclerosis are notable examples of degenerative spinal cord diseases. DCM is a common progressive disorder that can lead to quadriplegia. Macroautophagy, a cellular process responsible for the degradation of intracellular contents, has been implicated in many neurodegenerative diseases when disrupted. Smith et al. (2022) tested the hypothesis that macroautophagy is impaired in DCM using a series of human postmortem cervical spinal cord samples encompassing seven cases of DCM and five controls. They found that the number of LC3 puncta in the case group was significantly lower than that in the control group, consistent with a reduction in autophagy, and a large number of p62 aggregates was also observed in four of the seven cases, whereas none were detected in the control group. Additionally, in two of five cases assessed, tau levels were elevated relative to the controls, while Bcl-2 expression was also significantly higher in the case group (*P* = 0.0133), potentially explaining the decrease in autophagy. In more severe cases of DCM, a large number of Bcl-2 and p62 bodies were detected, suggestive of impaired autophagy. Moreover, greater autophagy impairment was correlated with damage severity.

ALS is the most common paralytic disease in adults, with disruption of the two major protein clearance pathways—the ubiquitin-proteasome system and autophagy—potentially playing a central role in its pathogenesis. Several ALS-linked mutations occur in genes encoding factors that are directly involved in protein degradation, including ubiquilin 2 and a mitochondrial protein, Coiled-coil-helix-coiled-coil-helix domain containing 10 (*CHCHD10*) (Taylor et al., 2016), both of which act as linkers, carrying polyubiquitinated proteins to the proteasome or autophagosome for degradation. Mutations have also been reported in optineurin, a proposed autophagy receptor and valine-containing protein, which plays a role in ER-associated degradation and the sorting of endosomal proteins. Other studies have reported frontotemporal dementia-associated mutations in charged multivesicular body protein 2B (*CHMP2B*), which encodes a protein implicated in autophagosome maturation and endosomal cargo sorting and degradation (Rusten and Simonsen, 2008; Xu et al., 2012; Lu et al., 2013).

### Autophagy-related epigenetic changes in spinal cord injury

#### DNA methylation regulates ATG expression

DNA methylation refers to the addition of a methyl group to the C-5 position of cytosine in the genomic CpG dinucleotide. This process is catalyzed by members of the DNA methyltransferase (DNMT) family, which includes DNMT1, DNMT3A, DNMT3B, and DNMT3L. The methylation of ATG16L, an autophagy-related gene, can inhibit the activation of autophagy and reduce medulloblastoma cell proliferation (Cruzeiro et al., 2018), and has been linked to multiple neurodegenerative disorders (Shu et al., 2023). The regulation of DNA methylation is associated with several neurological diseases, such as PD and AD. In PD, alkaline phosphatase methylation inhibits autophagy, resulting in alpha-synuclein aggregation. DNMT1 influences neurodegeneration via its regulatory effect on intracellular processes associated with protein homeostasis (Bayer et al., 2020). DNMT1 exerts a negative effect on autophagy and is involved in the clearance of proteins that are prone to aggregation by the aggregate-autophagy pathway (Wang et al., 2015b). In Huntington’s disease, DNMT1 knockdown enhances autophagy and ameliorates Huntington protein-induced cytotoxicity (Bayer et al., 2020). In SCI, meanwhile, alterations in DNA methylation patterns significantly impact the survival of neurons and astrocytes. In SCI animal models, the upregulation of DNMT1 results in hypermethylation at the promoter regions of the beclin-1- and LC3-encoding genes, thereby inhibiting autophagic flux and promoting neuronal death (Bai et al., 2024). A decrease in the level of non-CpG methylation after SCI can prevent the effective upregulation of the expression of key ATG proteins, thereby limiting autophagy. Consequently, damaged organelles, such as mitochondria, cannot be promptly cleared, resulting in the production of a large amount of ROS, which further damages neurons, exacerbates inflammatory reactions, disrupts the spinal cord microenvironment, and hinders neurological function recovery (Alirezaei et al., 2011).

#### Histone methylation

G9a/euchromatic histone lysine methyltransferase 2 (EHMT2) catalyzes the methylation of histone 3 at lysine 9 (H3K9me1, H3K9me2, and H3K9me3) and its expression is often elevated in a variety of cancers, including glioma and gastric and lung cancers (Yin et al., 2019; González-Rodríguez et al., 2021). G9a/EHMT2 overexpression has been significantly associated with autophagy inhibition. This methyltransferase can directly interact with the promoters of the core autophagy genes microtubule-associated protein 1 light chain 3 beta (*MAP1LC3B*), tumor protein p53 induced protein 2 dominant-negative (*TP53INP2*/*DOR*), and Wilson’s disease protein 1 (*WIPI1*), increasing their histone methylation levels (Artal-Martinez de Narvajas et al., 2013). The overexpression of EHMT2 has been found to inhibit autophagic activity in neurons at the lesion site in SCI, leading to increased neuronal death (Kim et al., 2020). Following SCI, the activation of EHMT2 was proposed to promote neuroinflammatory responses and exacerbate glial scar formation, thereby limiting axonal regeneration (Xu et al., 2021a). Another study on SCI showed that the EHMT2 inhibitor BIX-01294 can restore the expression of LC3B and beclin-1, increase autophagy levels, and reduce the expression of inflammatory cytokines at the injury site. Additionally, the use of EHMT2 inhibitors can promote axonal regeneration and functional recovery, indicating that EHMT2 might be a potential target for SCI treatment (Li et al., 2020b; Xu et al., 2021a).

Coactivator-associated arginine methyltransferase 1 (CARM1) is responsible for the demethylation of histone H3 at arginine 17 (H3R17me2). The AMPK-S-phase kinase-associated protein 2 (SKP2)/CARM1 signaling pathway is involved in autophagic transcriptional regulation and is associated with the pathology of a variety of diseases. In a nutrient-rich environment, CARM1 is typically degraded by the SKP2-containing SKP1/cullin1/F-box protein E3 ubiquitin ligase in the nucleus. Conversely, nutrient starvation activates AMPK-dependent forkhead box protein O3 (FOXO3) phosphorylation in the nucleus, which inhibits the transcription of SKP2, resulting in reduced CARM1 protein degradation (Shin et al., 2016). Elevated levels of CARM1 protein result in an increase in H3R17me2 on the promoters of autophagy-related and lysosomal-related genes, stimulating their transcriptional activation through a synergistic interaction with transcription factor EB (TFEB) (Zhou et al., 2020). Following SCI, ROS generation is enhanced, which impairs lysosomal function, ultimately leading to neuronal death. Meanwhile, an increase in the levels of transcription factor enhanced 3 (TFE3), partly mediated by the AMPK-SKP2-CARM1 signaling axis, can improve SCI outcomes. In addition, C9orf72, which is associated with ALS-frontotemporal dementia, can promote CARM1 lysosomal degradation, thereby influencing autophagy-lysosomal function and lipid metabolism (Zhou et al., 2020).

#### Post-translational regulation and autophagy-related diseases

Post-translational modifications, including the covalent attachment of novel functional groups such as phosphate, ubiquitin, methyl, and acetate moieties, are essential for protein activity, stability, folding, and interactions with other proteins. The inhibition of histone deacetylase-6, which is significantly upregulated after SCI, with tubastatin A enhances functional recovery following injury to the spinal cord. In contrast, its upregulation following SCI prompted acetylation and stabilization of microtubules, leading to autophagy inhibition and neuronal injury (Zheng et al., 2020). ULK1 protein is rich in phosphorylation sites, which allow the differential modulation of autophagy under different environmental conditions and influence its participation at different stages of autophagy. LPS induces the activation of p38 mitogen-activated protein kinase (MAPK), which subsequently phosphorylates the S757 and S504 sites of ULK1 in microglia, preventing it from binding to its downstream effector ATG13, resulting in decreased autophagy in these cells (He et al., 2018). Protein phosphatase 2A (PP2A) acts in several autophagy-related signal transduction pathways. Prolyl endopeptidase is a serine protease that has been shown to interact with PP2A as well as its endogenous inhibitor, recombinant *Arabidopsis thaliana* pectinesterase 1 (PME1), and phosphotyrosyl phosphatase activator (PTPA), resulting in the modulation of PP2A activity and levels. Prolyl endopeptidase inhibition reduces PP2A phosphorylation and increases its activity, leading to the phosphorylation of death-associated protein kinase 1 (DAPK1) and beclin-1, and thereby inducing autophagy. Several neurodegenerative diseases are associated with reduced PP2A activity, making PP2A activators promising candidates for pharmacological treatment of these conditions (Magnaudeix et al., 2013; Svarcbahs et al., 2020). One study showed that the activation level of PP2A is decreased post-SCI, leading to the dysregulated phosphorylation of beclin-1 and ULK1 and autophagic flux impairment (Luo et al., 2021). Meanwhile, Liu et al. (2015a) demonstrated that activating PP2A enhances autophagy levels after SCI and promotes injury repair. These findings underscore the therapeutic potential of regulating PP2A activity, ULK1 phosphorylation, or HDAC6 acetylation for SCI.

These insights into the molecular and epigenetic regulation of autophagy in SCI highlight potential therapeutic targets for enhancing autophagic activity and improving outcomes after SCI. Strategies targeting DNA and histone methylation and the modulation of post-translational modifications, such as HDAC6 inhibition and PP2A activation, may offer novel approaches for SCI treatment. Understanding the complex signaling pathways involved in autophagy regulation after SCI holds significant promise for developing more effective therapies to reduce neuronal damage, promote axonal regeneration, enhance functional recovery, and translate potential therapies into clinical applications.

## Crosstalk Between Other Modes of Cell Death and Autophagy in Spinal Cord Injury

### Crosstalk between apoptosis and autophagy

#### Apoptosis

As programmed cell death, apoptosis was first described by Kerr et al. (1972), who described different types of cell death in terms of morphology. Apoptosis is the process by which cells stop growing and dividing, ultimately leading to controlled cell death without spilling their contents into the surrounding environment (D’Arcy, 2019). It is a highly regulated process that cannot be stopped once it has begun. Apoptosis in SCI involves intrinsic and extrinsic pathways. The intrinsic pathway is triggered by mitochondrial dysfunction, which releases cytochrome c and activates caspase-9. The extrinsic pathway is initiated by death receptors (e.g., Fas/FasL), thus activating caspase-8. Both pathways converge on caspase-3, leading to DNA fragmentation and cell death. Apoptosis contributes to secondary injury, thereby exacerbating neuronal loss and functional deficits. Therefore, targeting apoptotic pathways might offer therapeutic potential for SCI treatment (Sobrido-Cameán and Barreiro-Iglesias, 2018; Shi et al., 2021b).

#### Apoptosis and autophagy

Autophagy and apoptosis are two essential and intricately connected mechanisms with critical roles in the regulation of cell fate. Their regulation involves many common signaling factors, such as p53, Ser/Thr kinases, BH3-only proteins, and oncogenes (Zhou et al., 2017; Fan et al., 2022). In the context of SCI, the interplay between these processes is critical for determining neuronal fate. For instance, phosphatase and tensin homolog (PTEN)-induced putative kinase 1 (PINK1) has been shown to collaborate with beclin-1 to initiate autophagosome formation in SH-SY5Y neuroblastoma cells, highlighting its anti-apoptotic properties (Brunelli et al., 2022). This PINK1-mediated autophagy protects neurons from apoptosis, possibly through its interaction with beclin-1. Conversely, the dysregulation of autophagy in SCI exacerbates apoptosis, contributing to neuronal loss (Jia et al., 2024).

Ca^2+^ also plays a pivotal role in SCI. Elevated Ca^2+^ levels promote autophagy via the Ca^2+^/calmodulin-dependent protein kinase kinase (CaMKKβ)/AMPK/mTOR signaling pathway, thus enhancing cell survival while simultaneously modulating apoptotic pathways. This dual role of Ca^2+^ in modulating both autophagy and apoptosis highlights the complexity of cellular responses after SCI. Additionally, lipid-protein interactions and specific plasma membrane microdomains (lipid rafts) modulate signaling pathways that govern cell proliferation, apoptosis, differentiation, and autophagy, all of which are critical in SCI pathophysiology (Sezgin et al., 2015, 2017; Liu et al., 2017; Gupta et al., 2021). ER lipid raft-associated proteins (ERLIN-1 and ERLIN-2) regulate cell survival and death by mediating ER-mitochondrial crosstalk, a process that is disrupted in SCI (Manganelli et al., 2021). Understanding the balance between autophagy and apoptosis in SCI can provide novel therapeutic targets to promote neuroprotection and repair. By modulating these pathways, it might be possible to enhance neuronal survival and reduce secondary damage following SCI.

#### Interaction between autography and apoptosis in spinal cord injury

**Figure 5:**
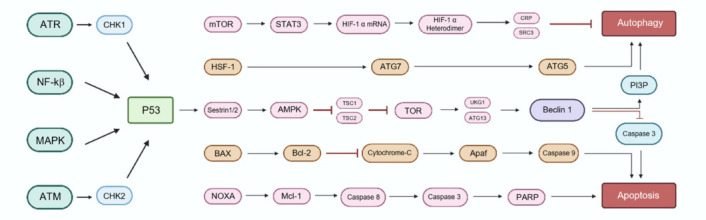
Cross-pathways of autophagy and apoptosis. Oxidative stress and DNA damage induced by spinal cord injury are the main causes of the upregulation of ATR, NF-κB, MAPK and ATM. They activate downstream signals through p53. The setrin1/2-Beclin-1 pathway inhibits apoptosis while promoting autophagy. AMPK: 5′ AMP-activated protein kinase; Apaf: apoptotic protease-activating factor; ATM: ataxia telangiectasia mutated; ATR: ataxia telangiectasia and Rad3-related protein; BAX: Bcl-2 associated X protein; Bcl-2: B-cell lymphoma 2; Caspase: cysteine-dependent aspartate-specific protease; CHK1: checkpoint kinase 1; CRP: calcium-binding protein; HIF: hypoxia-inducible factor; HSF: heat shock factor; MAPK: mitogen-activated protein kinase; Mcl-1: myeloid cell leukemia-1; mTOR: mammalian target of rapamycin; NF-kβ: nuclear factor kappa-light-chain-enhancer of activated B cells; NOXA: phorbol-12-myristate-13-acetate-induced protein 1; PARP: poly (ADP‒ribose) polymerase-1; PI3P: phosphatidylinositol 3-phosphate; SRC3: steroid receptor coactivator-3; STAT: signal transducer and activator of transcription; TOR: target of rapamycin; TSC: tuberous sclerosis.

Shi et al. (2021b) showed that the cysteine protease-3-mediated apoptotic pathway is activated after SCI. Other signaling pathways that trigger apoptosis after SCI include the Fas/FasR, sirtuin-1 (SIRT-1)/AMPK, Wnt/β-catenin, and E2F transcription factor 1/cyclin-dependent kinases signaling pathways, along with microRNAs and long noncoding RNAs. The PI3K/AKT/mTOR signaling pathway also induces apoptosis by activating the mitochondrial apoptotic pathway. In SCI, oxidative stress, elevated Ca^2+^ concentrations, impaired mitochondrial function, and ER stress are the main external signals that activate the cell death cascade, with BAX and Bcl-2 homologous antagonist/killer (BAK) serving as the key proteins in this process (Guo et al., 2023). Conversely, mTOR, tuberous sclerosis complex 1 (TSC1), and TSC2 are the main molecules involved in the activation of the autophagic pathway. In addition, ATG12 activates autophagosomes, cytochrome C, and caspases. The ubiquitin-proteasome system is a core mechanism underlying intracellular protein degradation. Initially, ubiquitin activation occurs through the ATP-dependent generation of a high-energy thioester bond between the cysteine residue at the active site of the ubiquitin-activating enzyme (E1) and the carboxyl terminus of ubiquitin. Furthermore, the mechanisms involved in the ubiquitin-proteasome system are extensively interconnected with those of autophagy and apoptosis, forming a complex regulatory network within the cells (Tsai et al., 2020). Enhanced expression of ubiquitin C-terminal hydrolase-L1 after traumatic injury impacts both autophagic and ubiquitin-proteasome system-related pathways. Working in concert, ubiquitin C-terminal hydrolase-L1 and trans-activator of transcription downregulate the expression of beclin-1 and LC3-II, thereby reducing autophagy and neuronal apoptosis (Liu et al., 2017). The p53 pathway is also an important molecular mechanism associated with both autophagy and apoptosis. Oxidative stress activates MAPK, NF-κB, ataxia telangiectasia and Rad3-related protein (ATR), and ataxia telangiectasia mutated (ATM) genes, leading to p53 pathway activation. In turn, p53 activates mTOR, heat shock factor 1, Sestrin1/2, BAX, and NOXA (phorbol-12-myristate-13-acetate-induced protein 1). mTOR and heat shock factor 1 are activators of autophagy, BAX and NOXA activate apoptosis, and Sestrin1/2 regulates both processes (**Figure 5**; Gupta et al., 2021).

In summary, the interplay between autophagy and apoptosis is essential for determining neuronal survival and death following SCI. Targeting key regulators of these processes could represent novel strategies for minimizing secondary damage and enhancing repair following SCI.

### Crosstalk between ferroptosis and autophagy Ferroptosis

Ferroptosis is a form of iron-dependent regulated necrosis that is driven primarily by iron-induced ROS accumulation and lipid peroxidation (Liu et al., 2020). Iron death is widely involved in a variety of pathological processes, including cancer and neurodegenerative diseases (Fan et al., 2022), and has been reported in animal models of traumatic SCI (Chen et al., 2020). In SCI, iron overload exacerbates oxidative damage, leading to membrane disruption, neuronal loss, and functional impairment. Iron toxicity enhances the Fenton reaction, which promotes ROS generation and damages key cellular components, including lipids, DNA, and proteins. Additionally, lipid peroxidation plays a critical role in iron-induced cell death, with a correlation between cell membrane disruption and lipid breakdown, which serves as a hallmark of this regulatory necrosis (Tang et al., 2019).

Lipid peroxidation involving polyunsaturated fatty acid phospholipids is catalyzed by specific enzymes, such as members of the acyl-coenzyme A synthase long-chain family, which are crucial in facilitating the death of cells through iron-induced mechanisms (Yang et al., 2016; Zhang et al., 2024). Among these, the arachidonate lipoxygenase (ALOX) family, comprising ALOXE3, ALOX5, ALOX12, ALOX12B, ALOX15, and ALOX15B, plays various roles in promoting iron-induced cell death, with distinct functions depending on the cell and tissue type (Liu et al., 2015b; Ma et al., 2022). Additionally, acyl-coenzyme A synthase long chain-4 also contributes to iron-induced cell death and may serve as a biomarker for this type of cell death (Yuan et al., 2016).

#### Ferroptosis in spinal cord injury

SCI is followed by a detrimental chain of events, characterized by excessive iron build-up combined with the inactivation of glutathione peroxidase 4 (GPX4), which ultimately gives rise to membrane damage and the death of neurons. Concurrently, during the occurrence of ferroptosis there are notable increases in both iron and ROS levels at the site of injury (Hao et al., 2017). The loss of GPX4 function has been linked to motor neuron degeneration and paralysis, as demonstrated in mouse models in which the conditional deletion of Gpx4 led to rapid neurodegeneration due to lipid peroxidation (Chen et al., 2015; Hu et al., 2017).

Therapeutic strategies targeting ferroptotic pathways have shown promising neuroprotective effects in SCI. Iron chelators, such as deferoxamine, have been reported to reduce ferroptosis-induced damage and improve functional recovery in SCI models (Ryan et al., 2024b). Additionally, GPX4 activation and lipid peroxidation inhibitors have demonstrated potential in mitigating ferroptosis and preserving neuronal function in experimental models of SCI (Kan et al., 2024).

Combined, these observations suggest that targeting ferroptosis offers potential therapeutic strategies for protecting neurons, reducing oxidative stress, and enhancing recovery following SCI.

#### Role of autophagy in ferroptosis

*Nuclear receptor coactivator 4-dependent ferritinophagy in spinal cord injury*

Nuclear receptor coactivator 4 (NCOA4)-dependent ferritinophagy involves the autophagic degradation of the major iron-storage protein in cells, ferritin, which is composed of a heavy chain (FTH1) and a light chain (FTL). In SCI, iron overload and oxidative stress are key contributors to secondary injury, leading to neuronal damage and functional impairment. NCOA4, identified as a cargo receptor in ferritinophagy, regulates iron homeostasis by mediating ferritin degradation. However, in SCI, excessive NCOA4 activity can lead to increased free iron levels, thereby exacerbating ferroptosis and neuronal damage (Gao et al., 2016; Liu et al., 2020; Li et al., 2021a).

In SCI, NCOA4-mediated ferritinophagy has been shown to significantly enhance iron-mediated oxidative stress, which further promotes neuronal damage. Increased levels of free iron not only stimulate lipid peroxidation, a hallmark of ferroptosis but also enhance ROS production, resulting in cell membrane rupture and neuronal loss. Studies employing SCI models have shown that the inhibition of NCOA4 or ATG proteins (e.g., ATG3, 5, 7, 13) can reduce the levels of free iron, alleviate oxidative damage, and protect against ferroptosis (Mancias et al., 2014; Kuno et al., 2022).

*Ras-related protein 7A-dependent lipophagy in spinal cord injury*

In cells, free fatty acids are esterified into triglycerides and cholesterol esters in the ER. This anabolic reaction leads to the accumulation of neutral lipids in microregions between the ER bilayer membranes, forming spherical organelles known as lipid droplets (Liu and Czaja, 2013; Liu et al., 2020). These intracellular lipid droplets are selectively targeted by autophagosomes for lysosomal degradation, a process known as lipid phagocytosis. Ras-related protein 7A (RAB7A) plays a crucial role in this context by facilitating the recruitment of lipid droplets to polyvesicles and lysosomes during lipid phagocytosis. Conversely, enhancing RAB7A-dependent lipid phagocytosis accelerates the degradation of lipid droplets, which, in turn, increases lipid peroxidation-induced ferroptosis (Bai et al., 2019; Lin et al., 2021). This suggests that targeting RAB7A-dependent lipophagy might help to balance lipid metabolism and reduce ferroptosis in SCI.

*Chaperone-mediated autophagy in spinal cord injury*

Heat shock protein family A member 8 (HSPA8), also known as heat shock cognate 71 kDa protein, is a constitutively expressed member of the HSP70 family. GPX4, a key ferroptosis inhibitor, is degraded via chaperone-mediated autophagy (CMA) in response to ferroptosis activation. This exacerbates ferroptosis and contributes to oxidative stress and neuronal damage or death in SCI. This process accelerates lipid peroxidation, which ultimately also leads to neuronal death (Chen et al., 2015; Hu et al., 2017).

In SCI, the loss of GPX4 significantly increases neuronal vulnerability to ferroptosis. It has been demonstrated that the ablation of GPX4 results in motor neuron degeneration and paralysis, underlining its essential role in protecting neurons from oxidative damage and ferroptosis (Liddell et al., 2024). HSPA5, a molecular chaperone related to ER stress, limits GPX4 degradation, thereby potentially protecting neurons from ferroptosis in SCI. HSPA5 helps mitigate the effects of oxidative stress by stabilizing GPX4 and reducing its turnover, which promotes cellular resilience (Zhu et al., 2017; Chen et al., 2021). Additionally, HSP90, another heat shock protein, was reported to enhance the stability of LAMP2A, a key receptor in CMA, which regulates GPX4 degradation during ferroptosis. By stabilizing LAMP2A, HSP90 enhances CMA, further protecting neurons from iron-induced oxidative damage and improving neuronal survival in SCI models (Wu et al., 2019; Liu et al., 2020).

*Beclin-1-mediated oxidative injury in spinal cord injury*

Beclin-1 was initially identified as a Bcl-2-binding protein through a yeast two-hybrid assay (Liang et al., 1998). Beclin-1 is a mammalian counterpart of yeast Vps30/ATG6, a key player in autophagy initiation and tumor suppression (Liang et al., 1998). The role of beclin-1 in regulating autophagy is particularly significant in SCI, where autophagy plays a crucial part in the secondary injury phase. In SCI, beclin-1 exerts both protective and detrimental effects, modulating oxidative stress and neuronal survival. Research has shown that the disruption of the beclin-1/SLC7A11 complex, which is crucial for glutamate/cysteine transport activity, can inhibit ferroptosis by preventing iron accumulation and lipid peroxidation. However, the activation of beclin-1 (e.g., via the Tat-beclin-1 peptide) may enhance lipid peroxidation (Kang et al., 2018; Song et al., 2018; Nagashima et al., 2022), which promotes ferroptosis and increases neuroinflammation in SCI. This dual role of beclin-1 in SCI is complex, as its activation may lead to neuronal damage while also providing a potential target for therapeutic strategies aimed at modulating autophagy and ferroptosis. In SCI, excessive beclin-1 activation can worsen secondary injury by increasing oxidative stress and promoting lipid peroxidation in neurons. Conversely, the inhibition of beclin-1 or the modulation of its interactions with SLC7A11 reduces the detrimental effects of ferroptosis and limits neuronal death. This suggests that fine-tuning beclin-1 activity may provide a novel therapeutic strategy for mitigating oxidative damage and enhancing recovery after SCI (Liu et al., 2025).

*High mobility group box-1 protein-mediated danger signals in spinal cord injury*

High mobility group box-1 protein (HMGB1), mostly localized to the nucleus, functions as a DNA-binding partner and is involved in the regulation of transcription, replication, and recombination. However, HMGB1 can also act as a damage-associated molecular pattern (DAMP) molecule, being either actively secreted by immune cells or passively released from dead or dying cells. HMGB1 also plays a position-dependent role in promoting autophagy. In the cytoplasm, HMGB1 binds to beclin-1, resulting in the induction of autophagosome formation (Tang et al., 2010; Liu et al., 2020). In SCI, HMGB1 release is triggered by ferroptotic activators such as erastin, sorafenib, and RSL3, highlighting its role in linking autophagy, ferroptosis, and secondary injury (Tan et al., 2024).

#### Interactions between autophagy and ferroptosis in spinal cord injury

Ischemia-reperfusion injury following SCI (SCIRI) is a serious condition that can result in severe sensory and motor dysfunction. Rong et al. (2022) showed that ferroptosis is a key pathological mechanism underlying SCIRI. Inhibition of ferroptosis has been demonstrated to aid in the recovery of motor function in mice post-SCIRI. Additionally, ubiquitin-specific peptidase 11 (USP11) is significantly upregulated in neuronal cells following hypoxia-reoxygenation and in the spinal cords of mice with ischemia-reperfusion injury. The knockdown of USP11 *in vitro* and its knockout *in vivo* (USP11-/Y) significantly reduced ferroptosis in neuronal cells. The overexpression of USP11 in mice resulted in increased ferroptosis and poor functional recovery after SCIRI. The upregulation of USP11 boosted autophagosome production and led to significant autophagic flux, potentially enhancing ferroptosis. Furthermore, reducing autophagy markedly diminished USP11-mediated ferroptosis, while inducing autophagy synergized with USP11 activity. Notably, ferroptosis enhances autophagy activation by stabilizing beclin-1 via USP11. The regulatory role of USP11 in ferroptosis has been elucidated through loss-of-function and gain-of-function studies, presenting new insights into both ferroptosis and autophagy.

Ferroptosis is a form of regulated necrosis driven by iron-induced ROS production and lipid peroxidation. This form of cell death plays a crucial role in various pathological conditions, including cancer and neurodegenerative diseases, and is increasingly recognized for its involvement in SCI. In SCI, iron overload exacerbates oxidative stress, leading to neuronal damage and functional impairment. Iron can also catalyze the generation of ROS, which can damage essential cellular components such as lipids, DNA, and proteins. Given the potential of therapeutic strategies targeting ferroptosis and autophagy, future research should focus on identifying specific molecular pathways involved in ferroptosis during SCI. Understanding the intricate balance among iron homeostasis, lipid metabolism, and autophagy could pave the way for the development of innovative treatments aimed at reducing neuronal damage and enhancing recovery during post-SCI. Additionally, strategies to modulate ferroptosis through iron chelation, GPX4 activation, and autophagy inhibition might offer novel avenues for protecting neurons and promoting functional recovery after SCI.

### Crosstalk between pyroptosis and autophagy

#### Pyroptosis

In 2001, the term “pyroptosis” combines the Greek root “pyro,” meaning fire or fever, with “ptosis” (from “to sis”), signifying a decline. This name aptly describes the inflammatory nature of this form of cell death, which is characterized by cell lysis and the release of proinflammatory cytokines. Pyroptosis serves as a defense mechanism against infection by rapidly rupturing the cell membrane and expelling intracellular pathogens into the extracellular space, where they are more susceptible to immune responses. Concurrently, pyroptosis triggers the release of proinflammatory cytokines and danger signals from affected cells, which attract additional immune cells to the site of infection, thus potentially facilitating the elimination of pathogens (Vande Walle and Lamkanfi, 2016; Hou et al., 2021). Once pathogens are activated, caspase-1 activates the inflammatory cytokine IL-1β. Processing the precursor of IL-18 into its active form leads to cell apoptosis while simultaneously releasing inflammatory cytokines into the surrounding environment (Wang et al., 2023c). Unlike apoptosis, nuclear integrity is maintained in pyroptosis (without fragmentation). Inflammasomes are typically assembled from NOD-like receptor family pyrin domain containing 1 (NLRP1) and NLRP3 sensor receptors, apoptosis-associated speck-like proteins with a caspase recruitment domain (ASC), and pro-caspase-1 (Stancu et al., 2019). Activating this inflammasome complex prompts the conversion of proinflammatory cytokines, specifically IL-1β and IL-18, from immature to active, secreted ones (Kesavardhana et al., 2020). Once activated, caspase-1 cleaves the gasdermin D protein to produce the bioactive gasdermin D-N, thus facilitating cell membrane perforation. This leads to the discharge of intracellular inflammatory mediators and initiates a cascade of inflammatory responses. In the context of SCI, pyroptosis plays a critical role in SCI by exacerbating inflammation and neuronal damage (McKenzie et al., 2020).

Pyroptosis in SCI is primarily mediated by caspase-1 or caspase-11/4/5, which cleave gasdermin D to produce its active fragment (gasdermin D-N). This fragment facilitates the formation of pores in the cell membrane, leading to pore formation, cell lysis, and the release of proinflammatory cytokines, such as IL-1β and IL-18. This process contributes to a proinflammatory environment that worsens SCI outcomes by promoting oxidative stress, inflammatory cell infiltration, and further neuronal damage (Dai et al., 2019; Al Mamun et al., 2021).

#### Pyroptosis and autophagy

Mitochondrial ROS (mtROS) play a crucial role in regulating pyroptosis, mainly through their influence on the NLRP3 inflammasome. In SCI, mitochondrial dysfunction leads to significantly elevated mtROS levels, followed by NLRP3 inflammasome activation and the subsequent induction of pyroptosis. This process exacerbates neuroinflammation and neuronal death, contributing to secondary injury (Zhu et al., 2017; Chen et al., 2025). This suggests that targeting mtROS may offer therapeutic benefits in SCI treatment. Recent studies have identified new agents that modify mtROS-associated cell death. SIRT-3, a key mitochondrial deacetylase, enhances mitochondrial autophagy, curbs ROS escalation, prevents NLRP3 inflammasome activation, and shields lipid-laden macrophages from stress.

The NF-κB-p62 mitochondrial autophagy pathway negatively modulates the activity of caspase-1, thus attenuating apoptosis and facilitating the interplay between autophagy and pyroptosis. Nuclear factor erythroid 2-related factor 2 (Nrf2), which is activated by p62, participates in the pyroptosis of macrophages. Overactivating and inhibiting the Nrf2/ARE signaling pathway can intensify or mitigate pyroptosis, respectively, indicating that p62 levels are adjusted through Nrf2 feedback mechanisms (Zhong et al., 2016; He et al., 2020). Meanwhile, Nrf2 inhibition can alleviate inflammation-mediated cell death in SCI. Furthermore, inhibiting autophagy was reported to enhance pyroptosis in macrophages treated with oxidized low-density lipoprotein via the p62/Nrf2/ARE pathway, presenting a novel therapeutic avenue for atherosclerosis. In SCI, similar mechanisms might exacerbate neuroinflammation and tissue damage. Targeting this pathway, for example, by enhancing autophagy or modulating p62/Nrf2 signaling, could provide a novel therapeutic strategy for reducing pyroptosis and improving outcomes in SCI.

#### Interactions between autophagy and pyroptosis in spinal cord injury

The activation of the cytoplasmic inflammasome complex, which leads to pyroptosis, is a critical event in neuroinflammation following secondary SCI (Xu et al., 2021b). Microglia are the immune cells most susceptible to pyroptosis after central nervous system injury, including in the spinal cord. Following such injuries, microglial activation triggers numerous signaling cascades that enhance the expression of the NLRP3 inflammasome, resulting in the pyroptosis of both microglia and neurons and the intensification of secondary damage (Broz and Dixit, 2016; Hu et al., 2020). Pyroptosis in microglia and neurons plays an important role during the secondary phase of SCI. Caloric restriction mimetics, such as 3,4-dichlorobenzonitrile (3,4-DC), enhance the beneficial effects of caloric restriction and exhibit therapeutic potential in neuroinflammatory conditions. Zhang et al. (2023a) demonstrated that 3,4-DC can diminish the glial scar area and reduce the death of motor neurons post-SCI, thereby aiding the recovery of spinal cord functions. RNA sequencing results revealed that oxidative stress, pyroptosis, necroptosis, and autophagy potentially contributed to the functional improvements observed with 3,4-DC treatment. Furthermore, 3,4-DC promotes autophagy by activating TFEB, an effect that is mitigated by TFEB depletion. Consequently, by boosting autophagy, 3,4-DC can inhibit both pyroptosis and necroptosis. Additionally, 3,4-DC modulates TFEB activity partially through the AMPK-transient receptor potential mucolipin 1 (TRPML1)-calcineurin signaling pathway. This regulatory mechanism highlights the multifunctional role of 3,4-DC in mitigating cellular stress and death pathways (Broz and Dixit, 2016).

### Crosstalk between parthanatos and autophagy

#### Parthanatos

Parthanatos, coined “par” and “Thanatos,” signifies a mode of cell death triggered by excessive poly(ADP-ribose) polymerase 1 (PARP-1) activation. The duration of parthanatos is correlated with swift PARP-1 activation, PAR-1 polymer synthesis, and mitochondrial depolarization. Notably, the translocation of nuclear apoptosis inducer factors in the early stages and potential late-stage caspase-related biochemical events are not obligatory for parthanatos, which do not rely on caspase activation for their progression. This trait distinguishes parthanatos from caspase-dependent apoptosis, as it remains impervious to pancaspase inhibitors. Unlike apoptosis, parthanatos does not yield apoptotic bodies or provoke minor DNA fragmentation; instead, it induces substantial DNA fragmentation. In SCI, overactivation of PARP-1 leads to NAD^+^ depletion, ATP loss, and mitochondrial dysfunction, resulting in apoptosis-inducing factor (AIF) translocation to the nucleus and DNA fragmentation. This process exacerbates neuronal death and inflammation, contributing to secondary injury in SCI. Therefore, targeting PARP-1 might offer therapeutic potential for SCI treatment.

#### Parthanatos and autophagy

Parthanatos is a form of programmed cell death driven by the overactivation of PARP-1, a DNA damage sensor, which leads to the production of excessive poly(ADP-ribose) (PAR). The accumulation of PAR in the cytoplasm triggers mitochondrial depolarization and facilitates the release of apoptosis-inducing factor from the mitochondria into the cytoplasm (Paddock et al., 2010; Park et al., 2020). In SCI, mitochondria are critically involved in secondary injury mechanisms, including the accumulation of ROS, which damages cellular structures and impedes recovery. In addition, given the essential role of mitochondria in the maintenance of cellular energy metabolism and homeostasis, their dysfunction during SCI accelerates neuronal loss. Simultaneously, mitophagy (mitochondrial autophagy) results in the selective targeting and degradation of damaged mitochondria. This process helps reduce ROS accumulation, preventing further damage to neurons and improving cellular survival (Lin et al., 2019; Schofield and Schafer, 2021). Given the role of mitochondria in SCI, a promising strategy for the treatment of SCI would involve not only mitigating mitochondrial damage but also scavenging ROS and preventing the build-up of toxic substances that contribute to neuronal death.

#### Interactions between autophagy and parthanatos in spinal cord injury

Research has highlighted the potential of zinc as a neuroprotective agent in SCI. Zinc has been shown to protect mitochondria and neurons, thereby fostering spinal cord recovery (Hu et al., 2021). Jiang et al. (2023) offered novel pre-clinical insights into the contribution of zinc to functional recovery after SCI. Oxidative stress following SCI engenders an abundance of ROS, which induces cell death through oxidative damage to proteins, lipids, and nucleic acids. Notably, oxidative DNA damage specifically triggers PARP-1 activation in parthanatos, with ROS accumulation playing a pivotal in its initiation. Mechanistically, the therapeutic effect of zinc may be achieved via the direct elimination of ROS and the alleviation of parthanatos through SIRT-3-mediated deacetylation of superoxide dismutase 2 (SOD2). Additionally, zinc may enhance mitochondrial autophagy via SIRT-3-β, which indirectly reduces ROS accumulation and alleviates parthanatos, thereby promoting neuronal survival and recovery after SCI.

The complex interactions among pyroptosis, parthanatos, and autophagy in SCI offer novel insights into therapeutic strategies targeting cell death and inflammation. The modulation of these pathways, including by enhancing the autophagy-mediated clearance of damaged mitochondria, inhibiting pyroptosis through inflammasome regulation, or targeting the molecular mechanisms underlying parthanatos, offers significant potential for improving outcomes in SCI. Emerging therapies targeting autophagy, mitochondrial function, and specific cell death pathways are likely to play a crucial role in mitigating secondary injury and promoting neuronal survival and repair post-SCI. Further research into the molecular crosstalk between these pathways is essential for developing effective treatments for SCI and other neuroinflammatory conditions.

### The interplay between mitochondrial autophagy and the cGAS-STING signaling pathway

The cGAS-STING signaling pathway, composed of cyclic GMP-AMP synthase (cGAS), STING, and downstream signaling adaptors, plays an essential role in immune defense against microbial DNA and cytosolic DNA associated with intracellular damage and is implicated in a range of immune diseases. In the cytoplasm, cGAS is responsible for recognizing foreign pathogens or damaged DNA. Following its binding to double-stranded DNA, cGAS recruits ATP and GTP and synthesizes cyclic guanosine monophosphate-adenosine monophosphate (cGAMP), which, in turn, binds to and activates the ER-resident protein STING (Shen et al., 2021). Activated STING translocates from the ER to the Golgi apparatus and induces the recruitment of IκB kinase complex β/IκB kinase (K1/IKK). TBK1 induces the production of interferon-I by promoting the dimerization of IRF3, a critical component of the immune response. IKK facilitates the nuclear translocation of NF-κB, thereby further modulating the immune response (Standaert and Childers, 2022).

Mitophagy is defined as the removal of defective or excess mitochondria via autophagy, a process that can be mediated by the PINK1/Parkin pathway, mitophagy receptors, or changes in mitochondrial lipid composition (Onishi et al., 2021; Jiménez-Loygorri et al., 2024). In the cytoplasm, mtDNA, due to its bacterial-like properties, may be mistaken for a foreign entity and has the potential to activate innate immune pathways, including the cGAS-STING axis (Li et al., 2023c).

The cGAS-STING pathway has been identified as a key mechanism in the neuroinflammatory processes contributing to AD pathogenesis, mediating tau protein-induced microglial activation and neuroinflammation (Xie et al., 2023). In PD, dopaminergic neurons in the substantia nigra are particularly vulnerable to inflammation. Chronic neuroinflammation driven by cGAS–STING activation exacerbates oxidative stress in these neurons, accelerates their degeneration, and leads to PD (Standaert and Childers, 2022). Multiple sclerosis is an autoimmune disease characterized by the loss of neuronal myelin sheaths. Chronic activation of the cGAS-STING pathway can amplify the inflammatory response in multiple sclerosis, thereby enhancing demyelination and neurodegeneration (Zhao et al., 2021b). This underscores the susceptibility of the neuronal and glial networks of the central nervous system to disruptions in homeostasis. Chronic activation of the cGAS-STING pathway leads to neuroinflammation, which adversely affects neuronal health, synaptic function, and overall brain integrity (Zhou et al., 2024).

## Autophagy Induction Improves Spinal Cord Injury

Many studies have shown that adequately inducing autophagy in SCI can lead to the clearance of damaged organelles and cells, delay cell death, promote injury repair, and maintain and restore neurological function (Lipinski et al., 2015). During the inflammatory response triggered by acute SCI, the regulation of mitochondrial function by astrocytes is associated with autophagy (Zheng and Tuszynski, 2023). Damaged mitochondria can be removed via autophagy, thereby maintaining the function of the mitochondrial network. In a mouse model of SCI with ATG7 knockout, clusters of overfused mitochondria were observed to form in astrocytes, followed by massive apoptosis (Motori et al., 2013). In the white matter, microglia and oligodendrocytes are the first cells to be affected during SCI.

In contrast, in grey matter, autophagosomes primarily accumulate in neurons. Notably, the function of motor neurons depends on autophagic flux (Liu et al., 2015a). mTOR, a negative regulator of autophagy, effectively reduces the phosphorylation of p70 ribosomal protein S6 kinase (p70S6K) and induces the upregulation of beclin-1 and LC3 expression in the SCI region. It also significantly reduces cell death in the spinal cord (Sekiguchi et al., 2012). Inhibiting the expression of beclin-1 using 3-methyladenine resulted in the suppression of autophagy and the promotion of apoptosis, leading to neuronal death (Tang et al., 2014; Feng et al., 2022). Similarly, neurons treated with the autophagy inducer rapamycin exhibited a significant increase in Bcl-2 expression, while neurons with high beclin-1 expression had higher LC3-II/LC3-I conversion rates, better cell viability, and lower levels of apoptosis (Wang et al., 2014). In a cell model of SCI, the induction of autophagy was shown to promote neurite growth, alleviate the inhibitory effects of nonpermissive substrate myelin, and reduce retraction sphere formation in cortical neurons following axonal injury. Furthermore, the induction of autophagy stabilized microtubules by degrading SCG101, a protein that mediates microtubule disassembly in neurons (Luo and Tao, 2020).

Similarly, in some animal models of SCI, therapeutic effects have been observed for both autophagy inducers and autophagy inhibitors. In a mouse model of acute traumatic SCI, the activation of autophagic flux reduced macrophage/neutrophil infiltration, microglia activation, and inflammation, while concomitantly increasing astrocyte proliferation and promoting neuronal survival and axon generation (Goldshmit et al., 2015; Ray, 2020). In acute and subacute rat models of traumatic SCI, treatment with valproic acid, an autophagy inhibitor, increased the expression of beclin-1 and LC3-II; decreased that of IL-1, TNF-α, and IL-10; and led to improvements in motor function (Wang et al., 2015a). Meanwhile, in a rat model of chronic traumatic SCI, valproic acid administration reduced autophagic cell death and prevented myelin damage in ventral horn motor neurons, thus promoting the recovery of motor function (Hao et al., 2013; Ray, 2020).

In summary, the primary role of autophagy activation in SCI is to prevent further damage, preserve the function of neural tissues, promote cell survival, and contribute to injury repair. There are various types of cells in the spinal cord, each with a distinct autophagy activation pathway. Neurons and astrocytes respond rapidly to autophagy activation under injury conditions and are also sensitive to the effects of autophagy impairment. Consequently, targeting autophagy and the expression of autophagy-associated proteins represents a potential new strategy for the repair of SCI.

## Clinical Translational Prospects for the Modulation of Autophagy in the Treatment of Spinal Cord Injury

Current treatments for SCI, including drug, surgical, and cell therapy, are limited to managing the injury status. Repairing damaged tissues and maximizing the restoration of neurological function remains a significant challenge. Notably, extensive methylprednisolone shock therapy does not improve the clinical symptoms of patients with SCI, instead increasing the occurrence of adverse effects (Karsy and Hawryluk, 2019; Wang et al., 2021a). The clinical outcomes of treatments, such as cell therapy and electrical nerve stimulation, are not satisfactory. Furthermore, severe treatment complications can significantly increase the suffering of patients, highlighting the need for alternative treatment strategies (Lai et al., 2021).

Autophagy can be considered a protective mechanism for neurons in the spinal cord under both physiological and pathological conditions. The important contributions of autophagy activation in SCI to the maintenance of cell survival, the degradation of functionally impaired components, and the delay of the onset of apoptosis are well documented. Accordingly, research efforts aimed at developing treatment strategies for traumatic SCI should focus on autophagy modulation. Pharmacological intervention targeting autophagy has the potential to not only control the progression of secondary SCI but perhaps also to enhance injury repair (**Additional Table 3**).

**Additional Table 3:** Agonists and inhibitors of autophagy in SCI

Type	Compound	Functionally relevant molecule	Signaling pathway or Key protein	Effect
Autophagic agonist	Metformin	LC3B, Beclin-1, p62	AMPK/mTOR	Activate autophagy, reduce tissue structure destruction, neuronal loss, and improve functional recovery after acute SCI
	FGF1	mTOR, Beclin-1, LC3-II/LC3-I	PI3K/Akt, MAPK/ERK, PRDX1	Activation of autophagy, neuroprotection, axonal regeneration and remyelination, anti–ROS effects
	NSCs-EVs	Beclin-1, 14-3-3t protein	mTOR related pathways	Activate autophagy, repair nerve damage, anti-apoptosis, anti-inflammation
	AS-IV	LC3B, Beclin-1, p62, CD16/32	mTORCl	Activate autophagy, inhibit neuronal apoptosis, suppress neuroinflammation, promote recovery of motor function
	BA	LC3II, Beclin1, Vps34	AMPK-mTOR-TFER	Activate autophagy, induce mitochondrial autophagy, and inhibit pyroptosis
Autophagy inhibitor	bpV	PTEN, LC3-II/LC3-I	Akt/mTOR	Autophagy inhibition, neuroprotection, functional recovery
	Co-ultraPEALut	Beclin-1, MAP-LC3, p62	PI3K/Akt/mTOR	Inhibit autophagy, improve neurobehavioral function, reduce apoptotic cell death and neuroinflammation, and improve tissue structure
	ABT888	Atg7, Atg5, Atg12, Beclin-1, LC3, p62, Bcl-2, Bax	PI3K/Akt/mTOR	Inhibit autophagy, anti-apoptosis, protects against SCI, and inhibit neuroinflammation
	3-MA	p62, LC3	PI3K pathway	Inhibit autophagosome production and protect neurons

3-MA: 3-Methyladenine; ABT888: veliparib; Akt: protein kinase B; AS-IV: Astragaloside IV; BA: baicalin; ERK: extracellular regulated protein kinase; FGF1: fibroblast growth factor 1; NSCs-EVs: neural stem cell extracellular vesicles; PRDX1: peroxiredoxin 2; PTEN: phosphatase and tensin homolog; Vps34: vacuolar protein sorting 34.

In conclusion, autophagy activation plays a key protective role in SCI by preventing further cellular damage, maintaining the survival of neurons and glial cells, and assisting in the repair process. Given the complexity of autophagic pathways in various spinal cord cell types, strategies targeting specific autophagy-related proteins may offer novel therapeutic opportunities for SCI treatment.

### Drugs that enhance autophagy

Metformin, an autophagy activator, reduces tissue structural damage and neuronal death and improves the recovery of neurological function after SCI (Zhang et al., 2017; Wang et al., 2020b). Metformin treatment was reported to increase the levels of LC3B and beclin-1 and reduce those of p62. Electron microscopic detection of autophagosomal vacuoles in neurons confirmed the presence of autophagy in these cells. Metformin can activate autophagy through the AMPK/mTOR pathway and aggravate SCI. In addition, fibroblast growth factor 1 (FGF1) treatment can lead to a significant reduction in mTOR phosphorylation levels and marked increases in beclin-1 and LC3-II/LC3-I expression levels, thus promoting autophagy after injury. FGF1 treatment also promotes axonal regeneration and remyelination, thereby enhancing tissue recovery, upregulates autophagy, and counteracts the effects of ROS by stimulating the expression of peroxiredoxin 1 (Li et al., 2018). Meanwhile, small extracellular vesicles derived from neural stem cells targeted 14-3-3t protein for delivery to cells. 14-3-3t subsequently activated autophagy through interactions with ULK1, raptor, and TSC2, ultimately promoting the recovery of spinal cord function. Furthermore, 14-3-3t overexpression was reported to exert an anti-apoptotic effect in rats with SCI (Rong et al., 2019). The compound astragaloside IV has been shown to inhibit mTORC1 in neuronal cells and microglia both *in vitro* and *in vivo*, thereby inhibiting both autophagy and apoptosis. Astragaloside IV also suppresses neuroinflammation by promoting the M2 polarization of microglia (Lin et al., 2020). Finally, betulinic acid was also reported to restore autophagic flux after injury via the AMPK-mTOR-TFEB signaling pathway and counteract ROS accumulation by inducing mitochondrial autophagy, thereby facilitating recovery after SCI (Wu et al., 2021).

### Drugs that inhibit autophagy

Bisperoxovanadium, an autophagy inhibitor, activates the AKT/mTOR signaling pathway, decreases the LC3-II/LC3-I ratio. Forelimb motor function was improved in mice with anterior cervical spinal cord contusion treated with bisperoxovanadium (Walker et al., 2012). ABT888, a novel PARP inhibitor, can also reduce the expression of the autophagosome formation-related proteins ATG7 and ATG5/12 as well as that of biomarker proteins for autophagy overactivation after SCI, resulting in injury amelioration. Similar ameliorative effects were observed with ABT888 treatment 24 hours after injury, reflected in significantly reduced histological injury and neutrophil infiltration and improved motor skills. ABT888 was proposed to exert its therapeutic effect through the PI3K/AKT pathway (Casili et al., 2020). Another autophagy inhibitor, 3-methyladenine, can reportedly inhibit PI3K and autophagosome formation, as well as reduce distal nerve degeneration (Bisicchia et al., 2017). In addition, astragaloside IV was shown to inhibit both autophagy and apoptosis in neuronal cells. To determine the effects of this compound on mTORC1 signaling *in vivo*, the authors of this study established SCI rat models and examined the ratio of p-mTOR/t-mTOR to p-p70S6k in spinal cord tissue by western blotting. They found that astragaloside IV treatment reduced mTOR and p70S6K phosphorylation levels and promoted microglial polarization, consequently suppressing neuroinflammation (Lin et al., 2020).

## Conclusion

Autophagy plays a crucial role in traumatic SCI, with both its induction and inhibition contributing to the response to injury. Research into autophagy-based therapies for SCI is ongoing. However, whether autophagy exerts protective or destructive effects depends on factors such as the degree and duration of damage. Both activation and inhibition of autophagy can be used in the treatment of traumatic SCI; accordingly, controlling autophagy levels after injury may represent an essential strategy for the rational use of autophagy activators and inhibitors. Nevertheless, the regulation of autophagy is highly complex, involving multiple signaling pathways, and achieving precise control over the degree and timing of autophagy remains a significant challenge.

Moreover, the local environment after SCI is often characterized by adverse factors such as inflammation, ischemia, and oxidative stress, which may interfere with the autophagy process. How to improve the local environment after SCI to support effective autophagy remains an important research goal. Autophagy is not only related to cell self-repair but is also interwoven with other cell repair mechanisms, such as apoptosis and necrosis. Excessive or insufficient autophagy can influence other cell death modalities, thereby affecting outcomes after SCI. We believe that indirectly regulating autophagy by regulating apoptosis may be a potential therapeutic strategy for this condition. However, how to coordinate autophagy with other repair mechanisms requires a better understanding of the molecular mechanism governing autophagy in different stages of SCI. Strict control over autophagy is essential to maximize its therapeutic potential. While some progress has been made in the use of autophagy regulators (such as mTOR inhibitors and agonists) in animal models, many challenges remain in translating these experimental results into clinical treatment. Issues such as drug delivery, dosage control, and side effects all need to be further verified in clinical trials.

In summary, although our understanding of the mechanisms underlying the involvement of autophagy in the repair of SCI remains incomplete, the therapeutic potential of autophagy, while still limited by a variety of factors, is undeniable. Future research should focus on how to precisely regulate autophagy to optimize its clinical use.

## Additional files:

***Additional Table 1:*** *Different types of microautophagy.*

***Additional Table 2:*** *Overview of the autophagic response after SCI.*

***Additional Table 3:*** *Agonists and inhibitors of autophagy in SCI.*

## Data Availability

*All relevant data are within the paper and its Additional files*.
